# Solid-Phase Synthesis as a Tool to Create Exactly Defined, Branched Polymer Vectors for Cell Membrane Targeting

**DOI:** 10.1021/acs.macromol.3c02600

**Published:** 2024-01-26

**Authors:** Johanna K. Elter, Veronika Liščáková, Oliver Moravec, Martina Vragović, Marcela Filipová, Petr Štěpánek, Pavel Šácha, Martin Hrubý

**Affiliations:** † Institute of Macromolecular Chemistry, CAS Heyrovského nám. 2, 162 06, Praha 6, Czech Republic; ‡ Institute of Organic Chemistry and Biochemistry, CAS Flemingovo nám. 2, 166 10, Praha 6, Czech Republic; § First Faculty of Medicine, Charles University Kateřinská, 1660/32, 121 08, Praha 2, Czech Republic

## Abstract

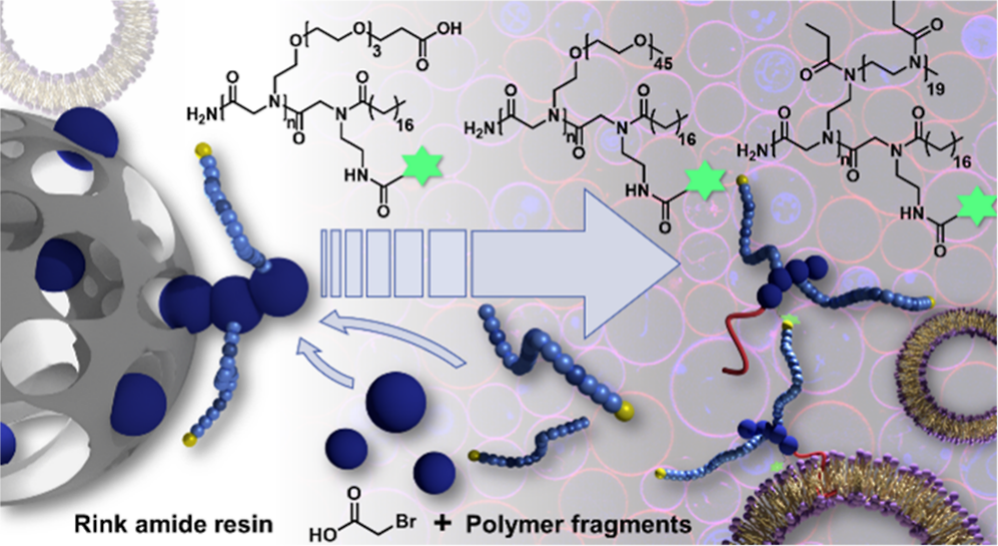

Modern drug formulations often require, besides the active drug molecule, auxiliaries to enhance their pharmacological properties. Tailor-made, biocompatible polymers covalently connected to the drug molecule can fulfill this function by increasing its solubility, reducing its toxicity, and guiding it to a specific target. If targeting membrane-bound proteins, localization of the drug close to the cell membrane and its target is beneficial to increase drug efficiency and residence time. In this study, we present the synthesis of highly defined, branched polymeric structures with membrane-binding properties. One to three hydrophilic poly(ethylene oxide) or poly(2-ethyloxazoline) side chains were connected via a peptoid backbone using a two-step iterative protocol for solid-phase peptoid synthesis. Additional groups, e.g., a hydrophobic anchor for membrane attachment, were introduced. Due to the nature of solid-phase synthesis, the number and order of the side chains and additional units can be precisely defined. The method proved to be versatile for the generation of multifunctional, branched polymeric structures of molecular weights up to approximately 7000 g mol^–1^. The behavior of all compounds towards biological membranes and cells was investigated using liposomes as cell membrane models, HEK293 and U251-MG cell lines, and red blood cells, thereby demonstrating their potential value as drug auxiliaries with cell membrane affinity.

## 1 Introduction

In the past decades, macromolecule-drug conjugates have received considerable attention in the field of drug development.^1−3^ Low solubility, premature decomposition, and rapid clearance rates from the bloodstream significantly lower the efficiency of small-molecular drugs.^4,5^ Furthermore, problems like insufficient biocompatibility and undesired side effects can occur.^6,7^ The covalent or noncovalent attachment of the drug molecule to a macromolecule enables fine-tuning of the aforementioned properties.^8,9^ The macromolecule can enhance the solubility of the drug by tuning the hydrophilicity of the conjugate.^10,11^ Decomposition, insufficient biocompatibility, and the occurrence of side effects can be addressed by shielding the drug against biological environments, e.g., via aggregation of the macromolecules and encapsulation of the drug within the core of the aggregate.^5,7^ Rapid renal clearance, which is typical for small-molecular drugs, can be avoided by creating a macromolecule-drug conjugate or an aggregate with a molecular weight above the renal threshold.^12^

Numerous molecular targets for different diseases are known in drug development, which opens up the possibility of designing specific drug molecules to activate or inhibit the latter. Many of these target receptors and enzymes are located on the surface of cells.^13,14^ Therefore, it is necessary to create strong interactions between the drug molecule and target to prevent rapid internalization into the cells or clearance of the substance from the site of action. Besides optimizing drug–target interactions, the optimization of the connected macromolecule can support localization on cell membranes. Structures with several reactive sites allow for the attachment of more than one drug molecule, which enables multivalent targeting on the cell surface. Tuning the distance between two drug molecules, or a drug molecule and another functional unit on the macromolecular carrier, permits detailed optimization of this process.^13,15,16^ By further incorporating a hydrophobic anchor into the polymeric structure, the polymer-drug conjugate can be localized close to the cell surface. Long-chain fatty acids, or cholesterol, can be used for this purpose, as these compounds can flexibly intercalate into cell membranes.^17,18^ This process can increase the local concentration of drug molecules in the area of the cell surface and extend the residence time of the drug at the site of action. Further, the attachment of a hydrophobic anchor may enable reversible binding of drug conjugates to proteins like albumin, which is another method to extend the half-life of a drug in the bloodstream.^19^ The hydrophobicity of the conjugate has to be exactly tuned to avoid micellization of the compound and unspecific attachment to cell membranes on the one hand and still achieve the desired effect via the combination of target affinity and cell membrane affinity of the conjugate.

Different types of macromolecules can be used to generate drug conjugates, e.g., (poly)peptides, synthetic polymers, or combinations thereof.^8,20,21^ Also, more complex biomacromolecules like antibodies can be used.^22,23^ Despite the ability of the latter to enhance the tissue specificity of the administered drug, they may cause molecule-specific side effects and their production is costly. (Poly)peptides and synthetic polymers are inexpensive and easy to produce on larger scales in comparison. While (poly)peptides are well-defined molecules that may as well exhibit advantageous tissue specificity and are further biodegradable, leaving behind harmless decomposition products,^20^ synthetic polymers exhibit a molecular weight distribution and a only partially defined sequence (e.g., the sequence of different blocks in block copolymers).^2^ In addition, not all synthetic polymers are biodegradable.^24^ On the other hand, polymers like poly(ethylene glycol)/poly(ethylene oxide) (PEG/PEO) exhibit the so-called stealth effect: besides being highly biocompatible, they adsorb a specific pattern of proteins in the bloodstream that leads to low recognition by the immune system, thereby preventing rapid clearance.^25,26^ High biocompatibility and low clearance rates can also be observed for certain poly(2-oxazoline)s, e.g., poly(2-methyl oxazoline) and poly(2-methyl oxazoline).^27−29^ The adsorption of specific proteins by these polymers was shown to be modulated by the topology and additional functional groups presented on the surface of the polymer coil or aggregate.^30,31^ Further, poly(2-oxazoline)s may be useful as immunomodulators.^32^ As PEG/PEO are reported to cause immune reactions in a growing number of individuals, poly(2-oxazoline)s may be promising candidates to replace PEG/PEO if needed.^33,34^ The combination of different synthetic monomers, e.g., ethylene oxide and glycidyl ethers or oxazolines, with different substituents in the 2-position allows for the introduction of a variety of functional groups into the polymer.^35−37^ Therefore, fine-tuning of the hydrophilicity and aggregation behavior of the molecule is possible.^38−40^ Additionally, reactive side and end groups can be used as linkers for the drug molecule or other functional units.^41^ For example, fluorescent dyes that are necessary for biological experiments or a hydrophobic anchor, as described before, can be incorporated. Attachment of these functionalities to peptides is possible as well, but the positions and reactive moieties that can be used are typically more restricted. Hence, the combination of structural aspects from both (poly)peptides and synthetic polymers may allow to combine the advantages of both types of macromolecular drug carriers.

Solid-phase synthesis (SPS) was developed for the synthesis of peptides by Merrifield in 1963.^42^ To this day, it has evolved into a common technique for the synthesis of different sequence-specific macromolecules. Besides (poly)peptides, peptide analogues like polypeptoids^43^ or polyamides from diacid derivatives and diamines can be generated.^44^ Oligonucleotides are mostly produced by means of solid-phase synthesis^45^ and some novel structural concepts of sequence-defined macromolecules have been introduced in recent years, e.g., oligo(thioether urethane)s for data storage applications by Du Prez et al.^46^ In medicinal chemistry, solid-phase synthesis can not only be applied to generate peptide-like drug molecules. If applied to the synthesis of drug carriers, the number and order of monomeric or polymeric building blocks connected within one macromolecule can be exactly defined. Drug molecules, additional targeting moieties, and functional units like fluorescent dyes can be introduced at the desired place in the chain—the introduction of an exact number of functional units is not restricted to the end groups of the macromolecule. This toolbox-like approach makes solid-phase synthesis a valuable tool for the generation of drug carriers.

In this work, solid-phase synthesis was applied to generate well-defined oligopeptoids with oligo(ethylene glycol) (OEG), PEG, or poly(2-ethyl oxazoline) (PEtOx) side chains. The structures show potential as macromolecules for polymer-drug conjugates. A two-step iterative synthesis approach was used to create OEG-, PEG-, or PEtOx-*N*-substituted glycines.^43^ Short OEG fragments with a defined number of repetition units are commercially available, and the oligopeptoids generated from OEG are monodisperse. The side chains contain carboxylic acids as end groups, which allows for further functionalization with one or more drug molecules. Further, we introduced a unit for postpolymerization modification with a cyanine 5 (cy5) dye.^47^ In the last synthesis step, acylation with stearic acid was carried out in order to introduce a hydrophobic end group to the molecule. This end group allows for locating the polymer drug conjugate close to cell membranes.^48^ To investigate the influence of the PEG chain length on the behavior of our structures, we further synthesized oligopeptoids containing polymeric PEG with a molecular weight of 2000 g mol^–1^ as side chains. Further, systems with poly(2-ethyloxazoline) (PEtOx, 2000 g mol^–1^) side chains were generated in order to study differences that are induced by the structure of the side chains.^49,50^ The application of different polymers further demonstrates the versatility of SPS as a method to generate branched or comb-like polymers. SPS has the potential to combine different synthetic polymers that cannot be connected in the same, well-defined way by, e.g., block copolymerization or conventional postpolymerization modification.

All products were characterized via suitable chromatographic methods and ^1^H NMR spectroscopy. The behavior of the compounds in aqueous solutions was studied using different techniques. We used both liposomes as artificial cell membranes^51−53^ as well as HEK293 and U251-MG cell lines to demonstrate that peptoid-based polymers with a hydrophobic anchor are able to attach to lipid bilayer membranes, which makes them promising candidates for conjugates that are supposed to target membrane-bound proteins. Cytotoxicity and hemolysis assays were conducted to determine suitable concentrations for the compounds to be used in *in vitro* cell experiments without significantly decreasing the metabolic activity of the cells or damaging the cell membranes.^54,55^

## 2 Experimental Section

### 2.1 Materials and Methods

Dry *N*,*N*-dimethylformamide (DMF) and dry dichloromethane (DCM) were purchased from Sigma-Aldrich Ltd. (Prague, Czech Republic). All other solvents were purchased from Lachner Ltd. (Neratovice, Czech Republic) and were of analytical grade.

Chloroform-*d* and MeOD were purchased from Eurisotop (Cambridge, U.K.). Amino-PEG_4_-*tert*-butyl ester was purchased from BroadPharm (San Diego, USA). Amino-PEG_45_-methyl ether was purchased from Iris Biotech (Marktredwitz, Germany). All other chemicals were purchased from Sigma-Aldrich Ltd. (Prague, Czech Republic). Acetonitrile, 2-ethyl-2oxazoline, and methyl tosylate used in the synthesis of poly(2-ethyl oxazoline) fragments were dried over CaH_2_ or BaO under argon, distilled, and stored over 4 Å molecular sieves prior to use. All other chemicals were used as received.

Sephadex-LH20 and Sephadex-G10 were purchased from Cytiva via Sigma-Aldrich Ltd. (Prague, Czech Republic) and equilibrated in either methanol (MeOH) or Millipore water for 3 h before packing in a gravity-driven separation column.

Proton nuclear magnetic resonance (^1^H NMR) measurements were performed on a 400 MHz Bruker AVANCE Neo spectrometer using CDCl_3_ or MeOD as a deuterated solvent. For calibration, the specific signals of the nondeuterated species were used.

Matrix-assisted laser desorption ionization–time-of-flight mass spectrometry (MALDI-TOF MS) mass spectra were acquired with the UltrafleXtreme TOF–TOF mass spectrometer (Bruker Daltonics, Bremen, Germany) equipped with a 2000 Hz smartbeam-II laser (355 nm) using the positive ion reflectron mode. Panoramic pulsed ion extraction and external calibration were used for molecular weight assignment. The dried droplet method was used in which solutions of the sample (10 mg mL^–1^) and the matrix (DHB, 2,5-dihydroxybenzoic acid, 20 mg mL^–1^) in methanol are mixed in a volume ratio 4:20. 1 μL of the mixture was deposited on the ground-steel target.

High-performance liquid chromatography (HPLC) measurements were carried out on a Dionex UltiMate 3000 UHPLC chromatograph (ThermoFisher Sci, USA) equipped with an RS pump module, a diode array detector (200/256/360/650 nm), and a fluorescence detector (fluorescence traces are not used). A Chromolith HighResolution RP18-e column with a water/acetonitrile eluent gradient (95% H_2_O/4.9% acetonitrile/0.1% trifluoroacetic acid (TFA) to 5% H_2_O/94.9% acetonitrile/0.1% TFA, 15 min) at a flow rate of 1 mL min^–1^ was used.

Gel permeation chromatography (GPC) measurements were carried out on a Dionex UltiMate 3000 UHPLC chromatograph (ThermoFisher Sci, USA) equipped with an autosampler, an UV–VIS detector (323 nm), an Optilab rEX rdifferential refractometer, and a DAWN 8+ multiangle light scattering detector (Wyatt; Santa Barbara, CA, USA). A TSK SuperAW3000 column with methanol and sodium acetate buffer (pH 6.5, 80/20 v/v) as an eluent at a flow rate of 0.5 mL min^–1^ was used.

### 2.2 Synthesis

#### 2.2.1 Synthesis of Amine-Terminated Poly(2-Ethyloxazoline)^56,57^

2-Ethyl-2-oxazoline (2 mL, 19.8 mmol) was dissolved in 5 mL of dry acetonitrile in a microwave vial in a glovebox. Then, methyl tosylate (152 μL, 1.007 mmol) was added, the vial was sealed, and it was placed in a heating bath that was preheated to 100 °C. The solution was stirred at 100 °C for 4 h before the polymerization was quenched by the addition of 300 mg (4.61 mmol) of solid sodium azide in the glovebox. Stirring at 100 °C was continued for 24 h. Then, the solution was cooled to room temperature, the reaction vessel was removed from the glovebox, solid residues were filtered off, and the solvent was removed to yield 1.9 g (0.95 mmol) of the crude polymer.

^1^H NMR (400 MHz, CDCl_3_): 3.75–3.40 (s, 80H), 3.16 (s, 1H), 2.61–2.33 (m, 40H), 1.26 (t, *J* = 15.9 Hz, 60H), *M*_n_ (^1^H NMR): 2100 g mol^–1^, *D̵*: 1.06.

Two methods were applied to convert the terminal azide group into an amine group for the purpose of using the polymer in the displacement reaction in solid-phase synthesis, as described in the following paragraphs.

##### 2.2.1.1 Method A^58^

Poly(2-ethyl-2-oxazoline) (1.9 g, 0.95 mmol) and 10% Pd on active charcoal (200 mg) were dissolved/suspended in 15 mL of dry MeOH under argon. Then, triethylsilane (1 mL, 10 mmol) was added dropwise to generate H_2_ in situ. After addition, the mixture was stirred at room temperature for 1 h. The major part of Pd/C was removed via filtration, and the solvent was removed under reduced pressure. Residual Pd/C was removed via dialysis in micropure water using a Spectra/Por regenerated cellulose dialysis tube with a molecular weight cutoff of 10,000 g mol^–1^. The solution surrounding the dialysis tube was collected, and the solvent was removed under reduced pressure.

##### 2.2.1.2 Method B^59^

Poly(2-ethyl-2-oxazoline) (1.9 g, 0.95 mmol) was dissolved in 20 mL of dry tetrahydrofuran (THF) under argon. The solution was cooled in an ice bath while 800 mg (3 mmol) of triphenylphosphine were added. The cooling was removed, and the solution was stirred at room temperature for 6 h. Then, 4 mL of micropure water were added, and stirring was continued overnight. Afterward, THF was removed under reduced pressure, additional water was added, and the solution was kept at 4 °C for 1 h before the precipitate of triphenylphosphine and triphenylphosphine oxide was removed via filtration. The solvent was removed under reduced pressure, and the product was purified on a Sephadex-LH20 column in MeOH.

^1^H NMR (400 MHz, CDCl_3_): 3.75–3.40 (s, 80H), 3.16 (s, 1H), 3.03 (t, *J* = 8.0 Hz, 2H) 2.61–2.33 (m, 40H), 1.26 (t, *J* = 15.9 Hz, 60H), *M*_n_ (^1^H NMR): 2100 g mol^–1^, *D̵*: 1.07.

#### 2.2.2 Synthesis of Compounds with Short PEG Chains ((PEG_4_COOH)_*X*_-amine-anchor)

Rink amide resin (330 mg, loading 0.6 mmol g^–1^, 0.2 mmol) was swollen in 2 mL of dry DMF for 10–15 min in a reactor with argon and vacuum connection. Then, removal of the Fmoc protective group was carried out using a 20% (v/v) solution of piperidine in dry DMF. The resin was reacted with 2 mL of the solution for 2 min and with further 2 mL of the solution for 15 min. Afterward, the resin was washed with dry DMF for 1 min three times.

Subsequently, bromoacetylation was carried out by adding a solution of 330 mg (2.37 mmol) of bromoacetic acid in 3 mL of dry DMF and 360 μL (2.32 mmol) of *N*,*N*′-diisopropylcarbodiimide (DIC) to the reactor. The reaction was allowed to proceed for 1 h before the resin was washed with dry DMF, as explained above. Then, amine displacement was carried out by adding a solution of 600 mg (1.87 mmol) of H_2_N-PEG_4_-(CH_2_)_2_–COO*t*Bu in 3 mL of dry DMF to the reactor and allowing the reaction to proceed for 3 h. Afterward, the resin was washed with dry DMF, as explained above.

Depending on the desired number of PEG fragments, bromoacetylation and amine displacement were repeated 0–2 times, as described.

Another bromoacetylation step was carried out as described. Then, an amine linker was introduced by adding 270 mg (1.69 mmol) of *N*-Boc-ethylenediamine in 3 mL of dry DMF to the reactor and allowing the reaction to proceed for 2 h. After washing, as described above, the final acetylation step was carried out. The resin was transferred to a round-bottom flask, flushed with argon, and a solution of 510 mg (1.79 mmol) of stearic acid or 157 μL (2.73 mmol) of acetic acid in 3 mL of dry DMF and 270 μL (1.74 mmol) or 405 μL (2.61 mmol) of DIC were added to the resin. The reaction was stirred at 40 °C overnight. Then, the resin was washed with warm DMF and warm DCM to remove precipitated stearic acid and other reactants and side products.

Cleavage of the products from the resin as well as cleavage of the *tert*-butyl and Boc groups was carried out by placing the resin in 4 mL of a mixture of TFA and H_2_O (95:5 v/v) and stirring for 1 h at room temperature. The resin was then filtered off using a PP filter and washed with TFA/H_2_O (4 mL) and DCM (10 mL). The solution was concentrated under reduced pressure, dissolved or suspended in water, and freeze-dried to yield the crude products that were used for further functionalization.

The following crude products were obtained:

##### 2.2.2.1 **1a**, (PEG_4_COOH)_1_-amine-SA, 121 mg

^1^H NMR (400 MHz, CDCl_3_): δ 7.52 (2s, br, 2H), 4.79–4.10 (m, 4H), 4.02–3.65 (m, 20H), 3.37 (2s, br, 2H), 2.74 (t, *J* = 7.5 Hz, 2H), 2.61 and 2.33 (t, *J* = 15.2, 7.5 Hz, 2H), 1.70 (m, br, 2H), 1.60–1.12 (m, 28H), 1.01 (t, *J* = 6.9 Hz, 3H), MALDI-TOF MS: 711.459 g mol^–1^ [M + Na^+^], HPLC: *t*_R_ = 11.63 min.

##### 2.2.2.2 **1b**, (PEG_4_COOH)_2_-Amine-SA, 198 mg

^1^H NMR (400 MHz, CDCl_3_): δ 7.22 (2s, br, 2H), 4.88–4.11 (m, 6H), 4.11–3.55 (m, 38H), 3.42 (2s, br, 2H), 2.76 (t, *J* = 9.9 Hz, 4H), 2.64 and 2.42 (2t, *J* = 7.6 Hz, 2H), 1.71 (s, br, 2H), 1.53–1.27 (m, 28H), 1.02 (t, *J* = 6.8 Hz, 3H), MALDI-TOF MS: 1016.725 g mol^–1^ [M + Na^+^], HPLC: *t*_R_ = 11.10 min.

##### 2.2.2.3 **1c**, (PEG_4_COOH)_3_-Amine-SA, 246 mg

^1^H NMR (400 MHz, MeOD): δ 6.93–6.27 (m, 2H), 4.87–4.14 (m, 8H), 4.01–3.60 (m, 54H), 3.26 (2s, br, 2H), 2.69 (t, *J* = 6.1 Hz, 6H), 2.48–2.34 (2t, *J* = 7.2 Hz, 2H), 1.71 (s, br, 2H), 1.38 (d, *J* = 25.8 Hz, 28H), 1.02 (t, *J* = 6.8 Hz, 3H), MALDI-TOF MS: 1321.988 g mol^–1^ [M + Na^+^], HPLC: *t*_R_ = 10.82 min.

##### 2.2.2.4 **1d**, (PEG_4_COOH)_2_-Amine-AA, 154 mg

^1^H NMR (400 MHz, CDCl_3_): δ 7.08 (m, 2H), 4.44 (m, 6H), 4.07–3.63 (m, 38H), 3.34 (2s, 2H), 2.73 (t, *J* = 5.3 Hz, 4H), 2.38 and 2.20 (2s, 3H), MALDI-TOF MS: 770.457 g mol^–1^ [M + H^+^], 792.441 g mol^–1^ [M + Na^+^], HPLC: *t*_R_ = 6.90 min.

#### 2.2.3 Synthesis of Compounds with Polymer Chains ((mPEG_45_)_*X*_-amine-anchor or (mPEtO*x*_20_)_*X*_-amine-anchor)

Rink amide resin (100 mg, loading 0.6 mmol g^–1^, 0.06 mmol) was swollen in 2 mL of dry DMF for 10–15 min in a reactor with argon and vacuum connection, as well as the possibility to conduct reactions for longer time periods while stirring under argon without the need for a continuous argon flow. The removal of the Fmoc protective group was carried out using a 20% (v/v) solution of piperidine in dry DMF, as described before. Afterward, the resin was washed with dry DMF for 1 min three times.

Bromoacetylation was carried out by adding a solution of 180 mg (1.58 mmol) of bromoacetic acid in 2 mL of dry DMF and 240 μL (1.55 mmol) of DIC to the reactor. The reaction was allowed to proceed for 1 h before the resin was washed with dry DMF, as explained above. In the next step, amine displacement was carried out by adding a solution of 1 g (0.5 mmol, *M*_n_ = 2000 g mol^–1^) of mPEG_45_-NH_2_ or mPEtOx_20_-NH_2_ in dry DMF to the resin. The reaction was allowed to proceed for 4 h before the polymer solution was replaced with a freshly prepared one. Then, the argon flow was stopped, and the reaction was allowed to proceed for further 18 h under gentle stirring. After that, the resin was washed with dry DMF, as explained above. The removed polymer solutions were collected and concentrated under reduced pressure. The polymer was purified by passing it through a Sephadex-LH20 column in MeOH, subsequent precipitation in cold diethyl ether (Et_2_O), and drying under high vacuum. The polymer can then be reused in synthesis.

Depending on the desired number of polymer fragments, bromoacetylation and amine displacement were repeated 0–2 times as described.

Another bromoacetylation step was carried out as described. Then, an amine linker was introduced by adding 200 mg (1.25 mmol) of *N*-Boc-ethylenediamine in 2 mL of dry DMF to the reactor and allowing the reaction to proceed for 3 h. After washing, as described above, the final acetylation step was carried out. A solution of 340 mg (1.19 mmol) of stearic acid or 70 μL (1.22 mmol) of acetic acid in 2 mL of dry DMF and 180 μL (1.16 mmol) were added to the resin. The argon flow was stopped, and the reaction was stirred at 40 °C overnight. Then, the resin was washed with warm DMF and warm DCM to remove precipitated stearic acid and other reactants and side products.

The cleavage of the products from the resin as well as the cleavage of the Boc group was carried out as described above. The compounds were purified via a Sephadex-LH20 column in MeOH.

For polymers generated from mPEtOx fragments synthesized via method A (see above), a hydrophilic side product with a molecular weight of 2000 g mol^–1^ was found in the products obtained from solid phase synthesis. To remove this impurity, the products obtained after purification via the Sephadex-LH20 column were dissolved in THF (150 μL THF for 10 mg of polymer) and adsorbed onto Amberlite-XAD4 resin (50 mg of resin for 10 mg of polymer; the resin was previously washed with THF to remove low molecular weight polystyrene). The polymer solution was added to the resin, shaken for 10 min, and then 1.5 mL of micropure water were added. Shaking was continued for 6 h before the supernatant was removed, and fresh THF (2 mL) was added for desorption. Desorption was carried out by shaking the resin in THF for 24 h. Then, the resin was removed via filtration and washed with THF. The THF solutions were combined, and the solvent was removed under reduced pressure to obtain the purified product. If residual amounts of the impurity were detected after this step, or for compounds without hydrophobic anchor, the amount of used compound in follow-up experiments was adjusted according to the amount of attached cy5 dye, as the impurity did not contain a reactive site for dye attachment.

The following products were obtained:

##### 2.2.3.1 **2a**, (mPEG_45_)_1_-Amine-SA, 75 mg

^1^H NMR (400 MHz, CDCl_3_): δ 7.83 (2s, br, 2H), 4.85–4.06 (m, 4H), 4.00–3.55 (m, 220H), 3.48 (s, 3H), 3.32 (2s, 2H), 2.53 and 2.28 (t, *J* = 7.5 Hz, 2H), 1.69 (m, 2H), 1.50–1.23 (s, 28H), 0.98 (t, *J* = 6.8 Hz, 3H), *M*_n_ (^1^H NMR): 2900 g mol^–1^, *D̵* (GPC) = 1.19.

##### 2.2.3.2 **2b**, (mPEG_45_)_2_-Amine-SA, 102 mg

^1^H NMR (400 MHz, CDCl_3_): δ 7.70 (2s, br, 2H), 4.72–3.97 (m, 6H), 3.95–3.48 (m, 451H), 3.45 (s, 6H), 3.26 (2s, br, 2H), 2.58, 2.53, 2.27, and 2.24 (4t, *J* = 6.8 Hz, 2H), 1.61 (s, br, 2H), 1.45–1.10 (m, 28H), 0.95 (t, *J* = 6.7 Hz, 3H), *M*_n_ (^1^H NMR): 5000 g mol^–1^, *D̵* (GPC) = 1.06.

##### 2.2.3.3 **2c**, (mPEG_45_)_3_-Amine-SA, 110 mg

^1^H NMR (400 MHz, CDCl_3_): δ 7.41 (2s, br, 2H), 5.01–4.04 (m, 8H), 4.02–3.54 (m, 674H), 3.51 (s, 9H), 3.27 (2s, br, 2H), 2.44–2.24 (m, 2H), 1.70 (s, br, 2H), 1.49–1.33 (m, 28H), 1.01 (t, *J* = 6.8 Hz, 3H), *M*_n_ (^1^H NMR): 7100 g mol^–1^, *D̵* (GPC) = 1.03.

##### 2.2.3.4 **2d**, (mPEG_45_)_2_-Amine-AA, 67 mg

^1^H NMR (400 MHz, CDCl_3_): δ 7.56–7.25 (2s, br, 2H), 4.82–4.03 (m, 6H), 3.74 (m, 462H), 3.48 (s, 6H), 3.28 (2s, 2H), 2.43–1.97 (several s, 3H), *M*_n_ (^1^H NMR): 4700 g mol^–1^, *D̵* (GPC) = 1.15.

##### 2.2.3.5 **3a**, (mPEtO*x*_20_)_1_-Amine-SA, 46 mg

^1^H NMR (400 MHz, CDCl_3_): δ 6.02 (s, br, 2H), 4.79–3.73 (m, 4H), 3.48 (m, 82H), 3.13 (s, 3H), 2.97–2.70 (m, 2H), 2.95–2.70 (m, 2H), 2.65–2.21 (m, 40H), 1.68 (m, 2H), 1.35 (s, 32H), 1.29–1.09 (m, 60H), 0.97 (t, *J* = 6.8 Hz, 3H), *M*_n_ (^1^H NMR): 2900 g mol^–1^, *D̵* (GPC) = 1.08.

##### 2.2.3.6 **3b**, (mPEtOx_20_)_2_-Amine-SA, 65 mg

^1^H NMR (400 MHz, CDCl_3_): δ 6.08 (s, 2H), 4.75–3.99 (m, 6H), 4.01–3.23 (m, 162H), 3.10 (s, 6H), 2.94–2.67 (m, 2H), 2.62–2.17 (m, 80H), 1.59 (m, 2H), 1.45–1.26 (m, 32H), 1.18 (m, 120H), 0.94 (t, *J* = 6.8 Hz, 3H), *M*_n_ (^1^H NMR): 5000 g mol^–1^, *D̵* (GPC) = 1.20.

##### 2.2.3.7 **3c**, (mPEtOx_20_)_3_-Amine-SA, 50 mg

^1^H NMR (400 MHz, CDCl_3_): δ 6.04 (s, 2H), 4.78–3.97 (m, 8H), 3.80–3.26 (m, 242H), 3.11 (s, 9H), 2.91 (m, 2H), 2.64–2.22 (m, 129H), 1.65 (m, 2H), 1.45–1.29 (m, 32H), 1.27–1.10 (m, 180H), 0.96 (t, *J* = 6.8 Hz, 3H), *M*_n_ (^1^H NMR): 7100 g mol^–1^, *D̵* (GPC) = 1.18.

##### 2.2.3.8 **3d**, (mPEtOx_20_)_2_-Amine-AA, 30 mg

^1^H NMR (400 MHz, CDCl_3_): δ 6.67 (s, br, 2H), 4.34–3.77 (m, 6H), 3.57 (m, 160H), 3.16 (s, 3H), 3.07 (m, 2H), 2.46 (m, 80H), 2.16 (s, 3H), 1.39–1.08 (m, 120H), *M*_n_ (^1^H NMR): 4700 g mol^–1^, *D̵* (GPC) = 1.10.

#### 2.2.4 Synthesis of cy5-MT Active Ester (3,3-Dimethyl-1-(6-oxo-6-(2-thioxothiazolidin-3-yl)hexyl)-2-((1*E*,3*E*)-5-((*E*)-1,3,3-trimethylindolin-2-ylidene)penta-1,3-dien-1-yl)-3*H*-indol-1-ium chloride)^47^

##### 2.2.4.1 1,2,3,3-Tetramethyl-3*H*-indol-1-ium Iodide

2,3,3-Trimethylindolenine (1 g, 6.28 mmol) and methyl iodide (900 μL, 9.42 mmol) were dissolved in 20 mL of dry acetonitrile in a microwave vial, sealed, and heated to 85 °C for 2 days. Then, the solvent was removed under reduced pressure, and the product was precipitated in cold Et_2_O. The obtained crystals were washed with additional cold Et_2_O to yield 1.181 g (6.21 mmol) of slightly pink crystals.

^1^H NMR (400 MHz, CDCl_3_): δ 7.84–7.77 (m, 1H), 7.74 (dd, *J* = 5.8, 3.3 Hz, 2H), 7.72–7.67 (m, 1H), 4.44 (s, 3H), 3.27 (s, 3H), 1.82 (s, 6H).

##### 2.2.4.2 1,3,3-Trimethyl-2-((1*E*,3*E*)-4-(*N*-phenylacetamido)buta-1,3-dien-1-yl)-3*H*-indol-1-ium Iodide

1,2,3,3-Tetramethyl-3H-indol-1-ium iodide (500 mg, 1.66 mmol) and 430 mg (1.66 mmol) of *N*-(3-(phenylamino)allylidene)benzenaminium chloride^60^ were suspended in 12 mL of acetic anhydride and heated to 100 °C for 1 h. Then, the mixture was cooled to room temperature, the amount of solvent was reduced under a vacuum, and the resulting slurry was diluted with DCM. Afterward, the product was precipitated in cold Et_2_O. The precipitate was collected, and the Et_2_O solution was left at 4 °C for 18 h to precipitate the residual product. In total, 690 mg (1.46 mmol) of the product were collected as a purple-to-black solid.

^1^H NMR (400 MHz, CDCl_3_): δ 8.89 (d, br, *J* = 10.7 Hz, 1H), 8.38 (s, br, 1H), 7.81–7.58 (m, 7H), 7.38 (d, *J* = 7.1 Hz, 2H), 6.94 (d, *J* = 14.8 Hz, 1H), 5.90 (t, *J* = 12.3 Hz, 1H), 4.18 (s, 3H), 2.36 (s, 4H), 1.89 (s, 6H).

##### 2.2.4.3 6-(2,3,3-Trimethyl-3*H*-indol-1-ium-1-yl)hexanoate

6-Bromohexanoic acid (1.276 g, 6.54 mmol) was added to a solution of 2,3,3-trimethylindolenine (700 μL, 4.36 mmol) in 15 mL of dry acetonitrile. The vessel was sealed and flushed with argon, and the mixture was heated to 80 °C for 4 days. Then, the solvent was evaporated, and the residue was heated for 2 min in ethyl acetate (3 mL). The resulting solid was collected, and the procedure was repeated once with ethyl acetate and twice with acetone as solvent. Collection of the resulting solid and drying under vacuum yielded 500 mg (1.83 mmol) of the product as a pale pink, amorphous solid.

^1^H NMR (400 MHz, CDCl_3_): δ 8.07–7.99 (m, 1H), 7.91 (dt, *J* = 7.6, 4.0 Hz, 1H), 7.83–7.75 (m, 2H), 4.67 (t, *J* = 8.0 Hz, 2H), 2.48 (t, *J* = 7.2 Hz, 2H), 2.20–2.07 (quin, *J* = 8.0 Hz, 2H), 1.84 (dt, *J* = 14.8, 7.2 Hz, 2H), 1.75 (s, 6H), 1.68 (dt, *J* = 10.1, 7.6 Hz, 2H).

##### 2.2.4.4 cy5-Hexanoate

A solution of the products from step 2.2.3.1 (225 mg, 0.475 mmol) and step 2.2.3.3 (130 mg, 0.475 mmol) was dissolved in 4 mL of dry pyridine and flushed with argon. Then, the mixture was heated to 40 °C for 3 h. The solvent was removed, and the product was purified via flash column chromatography (0 to 12% MeOH in DCM) to yield 125 mg (0.259 mmol) of the desired product as a dark blue solid.

^1^H NMR (400 MHz, CDCl_3_): δ 8.21 (t, *J* = 12.1 Hz, 2H), 7.53–7.44 (m, 4H), 7.33 (t, *J* = 7.5 Hz, 2H), 7.24 (dd, *J* = 11.7, 8.1 Hz, 2H), 6.99 (t, *J* = 12.4 Hz, 1H), 6.46 (dd, *J* = 21.0, 13.6 Hz, 2H), 4.19 (t, *J* = 7.4 Hz, 2H), 3.82 (s, 2H), 2.56 (t, *J* = 6.9 Hz, 2H), 2.02–1.90 (m, 2H), 1.88 (s, 6H), 1.86 (s, 6H), 1.88–1.80 (m, signal overlay, 2H), 1.75–1.60 (m, 2H).

##### 2.2.4.5 cy5-MT Active Ester

Cy5-hexanoate (50 mg, 0.104 mmol) was dissolved in 8 mL of dry DCM. The solution was flushed with argon, cooled to 0 °C in an ice bath, and EDC·HCl (44 mg, 0.230 mmol), 4-DMAP (3 mg, 0.025 mmol), and 2-mercaptothiazoline (22 mg, 0.185 mmol) were added. The reaction mixture was stirred for 2 h. Then, 5 mL of 0.2 M HCl were added, and the phases were separated. The organic phase was washed with 5 mL of 1 M HCl and then with 5 mL of water, dried with Na_2_SO_4_, and filtered, and the amount of solvent was reduced under vacuum. Then, the product was precipitated in cold Et_2_O and collected via centrifugation to yield 60 mg (quant.) of the product as a blue solid. The reactive dye was stored under the exclusion of moisture at −20 °C until use.

^1^H NMR (400 MHz, CDCl_3_): δ 8.24 (t, *J* = 12.1 Hz, 2H), 7.59–7.44 (m, 4H), 7.36 (t, *J* = 7.5 Hz, 2H), 7.24 (dd, *J* = 15.6, 7.9 Hz, 2H), 6.93 (t, *J* = 11.3 Hz, 2H), 6.43 (dd, *J* = 13.4, 4.8 Hz, 2H), 4.72 (t, *J* = 7.5 Hz, 2H), 4.20 (t, *J* = 7.4 Hz, 2H), 3.83 (s, 3H), 3.47 (t, *J* = 7.5 Hz, 2H), 3.40 (t, *J* = 7.2 Hz, 2H), 2.21–2.09 (m, 2H), 2.02–1.94 (m, 2H), 1.89 (s, 6H), 1.87 (s, 6H), 1.76–1.61 (m, 2H).

#### 2.2.5 Attachment of the Dye to the Oligopeptoids

**Figure 1:**
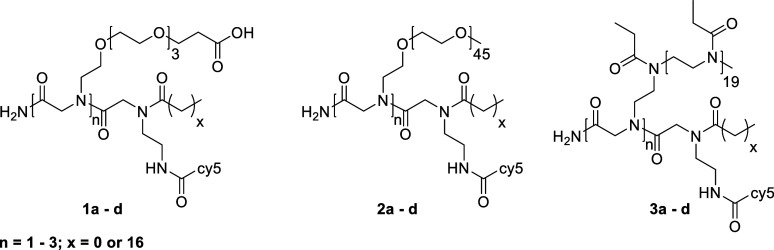
Final compounds based on functional OEG (1a–1d), mPEG (2a–2d), and mPEtOx (3a–3d).

**Figure 2:**
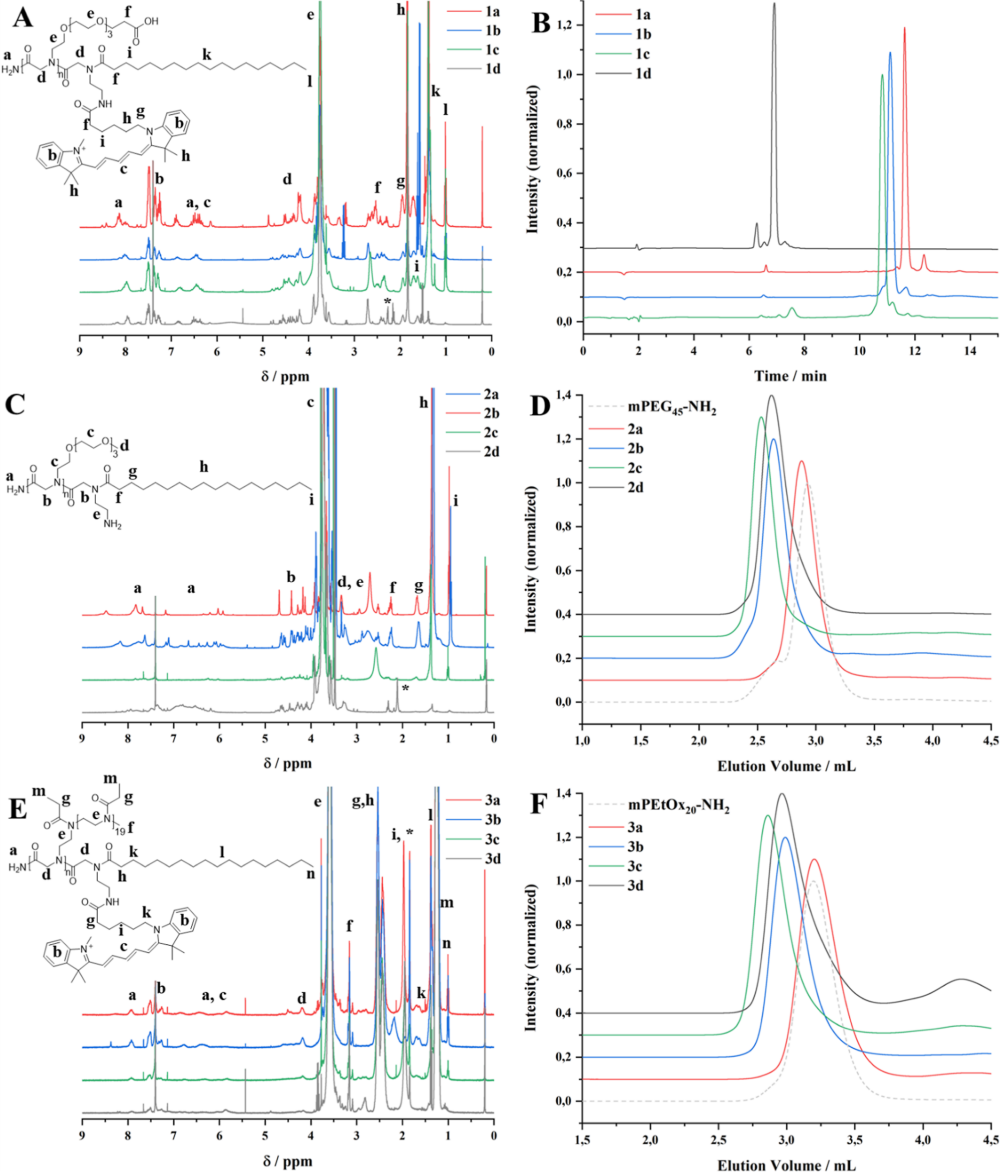
^1^H NMR spectra of compounds 1a–1d (A), 2a–2d, and (C) 3a–3d (E), HPLC traces of compounds 1a–1d (B), and GPC traces of compounds 2a–2d (D) and 3a–3d (F). ^1^H NMR spectra were recorded before freeze-drying for biological studies and can therefore contain solvent residues (e.g., EtOH). The ^1^H NMR spectra of compounds 2a–2d were recorded before the attachment of cy5. An exemplary ^1^H NMR spectrum after attachment of cy5 can be found in the Supporting Information (Figure S1).

10 mg of any product of 2.2.1 or 2.2.2 was dissolved in 2 mL of dry DCM, and the solution was flushed with argon. Then, 100 μL of DIPEA and 3 eq of cy5-MT active ester compared to the branched compounds were added, and the mixture was stirred in the dark for 18 h at room temperature. The products were purified via a Sephadex-LH20 column in MeOH. For compounds with small PEG chains, an additional purification step was performed. In this step, the product was dissolved in Millipore water and passed through a Sephadex-G10 column. The structures of the obtained products are depicted in Figure 1, and analytical data is shown in Figure 2.

MALDI-TOF MS for monodisperse compounds 1a–1d: 1a: 1153.890 g mol^–1^ [M^+^], 1b: 1459.097 g mol^–1^ [M^+^], 1c: 1764.251 g mol^–1^ [M^+^], 1d: 1234.768 g mol^–1^ [M^+^].

### 2.3 Investigation of Solution Structures

#### 2.3.1 Dynamic Light Scattering and Zeta Potential Measurements

Dynamic light scattering (DLS) measurements were performed on a Malvern Zetasizer Nano ZS instrument with a scattering angle of 173°. Samples were prepared by dissolving 1 mg of the nonlabeled oligopeptoid in 1 mL of micropure water for DLS measurements or in PBS (pH = 7.4) for Zeta potential measurements. The averaged intensity autocorrelation functions (ACF) of the DLS measurements were evaluated using the non-negative least-squares (NNLS) analysis implemented in the Zetasizer software, resulting in a distribution of sizes converted from distributions of diffusion coefficients using the Stokes–Einstein relation
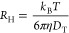
where *k*_B_ is the Boltzmann constant, *T* is the absolute temperature, and η is the viscosity of the solvent.

#### 2.3.2 Fluorescence Correlation Spectroscopy Measurements

For fluorescence correlation spectroscopy (FCS) measurements, solutions of the compounds with cy5 (0.1 μg mL^–1^) and without cy5 (1 mg mL^–1^) in micropure water were prepared. Then, the appropriate amount of the solution of the labeled compound was added to 100 μL of the corresponding solution of the unlabeled compound to obtain a final concentration of ca. 30 nM of the labeled compound within the solution. 30 μL of the solution were then transferred to a glass bottom Petri dish for the measurement. (Cellvis, Sunnyvale, California, USA). The measurements were performed using an Olympus IX83 confocal laser scanning microscope controlled with FluoView 1200 software (Olympus Corporation, Japan), extended with a FLIM/FLCS upgrade kit driven by SymphoTime64 software (PicoQuant GmbH, Germany). The fluorophore of the compounds was excited by an LDH-D-C-640 laser diode emitting 635 nm light, driven by a PDL 828 Sepia II driver in picosecond pulsed mode at a 40 MHz repetition rate (both devices: PicoQuant) through the 635 nm dichroic mirror built into the IX83 scan head. An Olympus UPlanSApo water immersion objective (60×, 1.2 NA) delivered the excitation light into a diffraction-limited spot and collected the emitted fluorescence. The laser intensity was maintained at approximately 20 μwatt average power at the objective entrance pupil to avoid photobleaching and/or saturation. The collected fluorescence passed through a Semrock 690/7 nm BrightLine emission filter and was detected by a hybrid photomultiplier (PMA Hybride-40 from PicoQuant) operated in photon counting mode. Photon counts were recorded using a PicoHarp300 TCSPC module in T3 time tagging mode. The SymPhoTime64, ver. 2.1 software from PicoQuant, was used for data acquisition and FCS data analysis. Each acquisition took 2 min on average, and the measurements were performed at 23 ± 1 °C. The FLCS ACF for the simplest case of one diffusing component is mathematically given by the equation
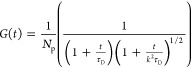
wherein *N*_p_ is the average number of diffusing fluorescent particles in the confocal volume, *t* is the correlation time, the diffusion time τ_D_ refers to the residence time of fluorescent objects in focus, and *k* is the ratio of axial to radial radii of the confocal volume, *k* = *w*_*z*_/*w*_*xy*_ with *w*_*xy*_ and *w*_*z*_ being the dimensions of the focal spot in the *x*–*y* plane (perpendicular to the optical axis) and along the *z*-axis. Then, the diffusion time can be expressed as τ_*D*_ = *w*_*xy*_^2^/4*D*_T_, where *D*_T_ is the coefficient of translational diffusion of the compounds or their aggregates. Diffusion coefficients were obtained by fitting measured ACFs with appropriate model functions, and hydrodynamic radii of the compounds or their aggregates in solution were subsequently obtained using the Stokes–Einstein equation (see DLS measurements).

#### 2.3.3 UV/Vis Measurements

UV/vis spectra of the cy5-labeled compounds were recorded on an Evolution 220 UV/vis spectrometer (Thermo Scientific, USA) using solutions of the compounds in micropure water (10 μg mL^–1^).

#### 2.3.4 Fluorescence Spectroscopy Measurements

Fluorescence excitation and emission maps were recorded on a FluoTime 300 instrument—fluorescence steady-state and lifetime spectrometer—using solutions of the compounds in micropure water (10 μg mL^–1^). The spectrometer was equipped with a PDL 820 computer-controlled driver (PicoQuant GmbH, Berlin, Germany). The cy5-labeled samples were placed in disposable cuvettes and were excited with a UV-xenon lamp operating in continuous-wave mode. Fluorescence emission (λ_em_ = 660 nm for excitation maps and λ_ex_ = 638 nm for emission maps were chosen after optimization) was recorded using high-resolution excitation and emission double monochromators and detected by a PMA hybrid photon detector. The measurement setup was automatically optimized and kept for each series of measurements. Data were analyzed using EasyTau software, version 2.2.3293 (PicoQuant GmbH, Berlin, Germany). All the measurements were performed at laboratory temperatures.

For CMC measurements, 4 mg (19.8 μmol) of pyrene were dissolved in 330 μL of acetone. Then, 10 μL of this solution were added to 50 mL of PBS (pH = 7.4) in a glass vial. Solutions of different concentrations (0.2–4 mM) of the compounds without cy5 in PBS were prepared in glass vials. Then, 500 μL of the pyrene solution were added to 500 μL of the compound solution for reach sample, shaken, and stored at 4 °C overnight. The fluorescence emission spectra (λ_ex_ = 339 nm, λ_em_ = 350–425 nm) of the compounds were recorded on the same device as the fluorescence excitation and emission maps of the compounds. The measurement setup was automatically optimized and kept for each series of measurements. For determining the CMC, the fluorescence emission of the samples for both λ_em_ = 372 nm and λ_em_ = 392 nm was plotted versus the decadic logarithm of the compound concentration in solution. A sharp increase in fluorescence emission with concentration can be detected above the CMC.

#### 2.3.5 Binding of the Conjugates to Artificial Membranes

For the investigation of the ability of the compounds to attach to lipid bilayer membranes, giant vesicles were prepared as cell membrane mimics. The lipid dioleoylphosphatidylcholine (DOPC, 5 mg mL^–1^) and the polymer poly(ethylene oxide)-*b*-1,2-poly(butadiene) (PEO-*b*-PBD, 1 mg mL^–1^) were dissolved in chloroform. For visualization of the vesicles, 5 mol % of PEO-*b*-PBD were labeled with the fluorescent dye atto390. 20 μL of the solution were spread on an indium tin oxide-coated (ITO) glass coverslip (VesiclePrepChamber, Nanion Technologies GmbH, München). The dried thin film was formed by the complete evaporation of chloroform at room temperature for approximately 2 h in a vacuum chamber. A 16 × 1 mm O-ring was placed on this coverslip, and another ITO-coated coverslip was placed on top. The space between the coverslips was filled with 250 μL of a 100 mM sucrose solution in micropure water to rehydrate the film. To make vesicles on the ITO-coated glass slide, Vesicle Prep Pro was used (Nanion, Germany). The protocol for making liposomes was set up for 1 h, 2 V, and 10 Hz. After the formation of the liposomes, a 200 mM solution of glucose (two to three times the volume of the liposome solution) was added in order to induce sedimentation of the liposomes. After 1 h, the settled liposomes were carefully collected from the bottom of the vessel. 100 μL of liposome solution was used for incubation with each of the peptoid compounds. The labeled peptoid was added to a 1 mg mL^–1^ stock solution in micropure water to adjust a concentration of 10 μmol L^–1^ in the samples. The samples were incubated for 3 h at room temperature before 500 μL of glucose solution were added to settle the vesicles and thereby separate them from the leftover peptoid in the solution.

For imaging, 30 μL of liposome solution were taken from the bottom of the vials used for sedimentation after the final washing step and transferred to glass bottom Petri dishes (Cellvis, Sunnyvale, California, USA). Confocal laser scanning microscopy (CLSM) experiments were carried out on an IX83 confocal laser scanning microscope (Olympus, Tokyo, Japan) using an Olympus 60× water immersion objective (UPLSAPO60XW NA:1.20). White light was used to detect the particles, while an UV (405 nm) and a red (635 nm) laser were used for fluorophore excitation. A dichroic mirror (DM405/488/543/635) was used to collect the emitted fluorescence. FluoView 1000 software version 4.2.3.6 (Olympus) was used for data acquisition and the generation of images.

For binding reversibility studies, 20 μL of the incubated and nonincubated liposome solutions used for imaging before, respectively, were transferred to a fresh glass bottom Petri dish and homogenized using an Eppendorf pipet. Images were taken directly after mixing (the liposomes settled after some minutes) as well as 2 h after mixing.

### 2.4 In Vitro Cell Assays

#### 2.4.1 Experimental Procedures on HEK293 and U251-MG Cell Lines

Cell binding affinity, uptake/localization studies, and the cell viability assay were performed on HEK293 cells (obtained as indicated in the acknowledgments), which were cultivated on a 100 mm Petri dish (Biofill, #TCD000100) with a cell density of 4 × 10^4^ cells cm^–2^. The cells were maintained in 10 mL of Iscove’s Modified Dulbecco’s Medium (IMDM; Biosera, #LM-I1090) supplemented with 10% fetal bovine serum (FBS; Sigma-Aldrich, #F7524), further referred to as IMDM complete.

The U251-MG cells (obtained from ATCC as U373-MG) were maintained in 10 mL of Dulbecco’s modified Eagle medium (DMEM; Sigma-Aldrich, #D6429) supplemented with 10% FBS, further referred to as DMEM complete. Both cell lines were maintained at 37 °C with a 5% CO_2_ atmosphere in an incubator. Experimental details on the incubation experiments are listed in Sections 2.4.2, 2.4.4, and 2.4.5.

#### 2.4.2 Cell Viability Assay

To probe the cytotoxicity of the substances, a CellTiter-Glo Luminescent Cell Viability Assay (Promega, #G7570) was conducted to quantify adenosine triphosphate (ATP) levels. Following the washing of HEK293 cells, cultivated as described above with PBS [137 mM NaCl (Lachner, #61013), 2.5 mM KCl (Lachner, #61012), 8.1 mM Na_2_HPO_4_·2H_2_O (Lachner, #30388), and 1.5 mM KH_2_PO_4_ (Lachner, #30016), pH = 7.4], the cells were collected and quantified using the Countess Automated Cell Counter (Invitrogen). Subsequently, the desired cell quantity was seeded into individual wells of a 384-well cell culture plate (Cellstar, Greiner Bio-One GmbH, #392-0311) in 20 μL of IMDM complete. A dilution series (10 μM, 2.5 μM, 625 nM, 156.3 nM, 39.1 nM, and 9.8 nM) of 5 μL of the different cy5-labeled compounds was added to the respective wells containing cells. Incubation periods of 3 h to probe immediate membrane toxicity or 72 h to investigate possible long-term damage of the cell membrane or toxic effects after uptake of the compounds at 37 °C with a 5% CO_2_ atmosphere were employed accordingly. After incubation, samples were subjected to analysis using an Infinite M1000 plate reader (Tecan) with the addition of CellTiter-Glo Luminescent Cell Viability Assay reagent in a 1:1 ratio, following the manufacturer’s protocol. Luminescence measurements were recorded after 3 and 72 h of incubation for real-time viability assessment. Each assay was performed with three technical replicates and three biological replicates. The relative viability values, presented as the mean ± standard deviation (SD), were normalized to the respective untreated control cells in each assay (3 and 72 h).

The cell viability assay for the U251-MG cell line was conducted following the aforementioned protocol with minor adjustments. The cytotoxicity experiment on U251-MG cells was conducted for 72 h and was carried out in DMEM complete, employing three technical replicates (one biological replicate). The relative viability values, normalized to the corresponding untreated control samples, are presented in the Supporting Information (Figure S6).

#### 2.4.3 Hemolysis Assay

The hemolysis assay was carried out to test the possible cell membrane-disruptive properties of the substances. For the experiment, fresh human whole blood was collected into ethylenediaminetetraacetic acid tubes and centrifuged at 423 × *g* for 3 min. Separated blood plasma was discarded, and sedimented red blood cells (RBCs) were washed 3 times with sterile PBS (pH = 7.4). After the last wash, the RBCs were counted and diluted to a concentration of 10^8^ cells per mL of PBS. 100 μL of this suspension were pipetted into each well of a 96-well plate, and the plate was centrifuged at 1500 × *g* for 10 min. The supernatant was removed, and the RBCs were mixed either with the cy5-labeled oligopeptoid compounds to yield final concentrations of 0.156–40 μM, with PBS as a negative control, or with 1% Triton X-100 (Sigma-Aldrich, St. Louis, MO, USA) as a positive control with full hemolysis. The samples were incubated at gentle shaking (300 rpm) for 1 h at 37 °C. After incubation, the plate was centrifuged, and 50 μL of the supernatant from each well was transferred into a new 96-well plate. The absorbance of released hemoglobin was measured at 540 nm using a Synergy H1 multimode reader (BioTek Instruments, Inc., Winooski, Vermont, USA). The relative hemolytic activity, presented as the mean ± SD, was expressed in comparison to the positive control (1% Triton X-100). Healthy donor blood was acquired from the Military University Hospital Prague from patients of the facility according to the availability of residual samples.

#### 2.4.4 Flow Cytometry

Interactions and binding of all presented compounds with HEK293 cells were investigated using flow cytometry. For this purpose, the HEK293 cells, cultivated as described above, were rinsed with PBS, collected, and resuspended in IMDM complete. The cells were then diluted to a concentration of 1 × 10^6^ cells mL^–1^. Afterward, 100 μL of the cell suspension was dispensed into separate wells of a polypropylene 96-well U-shaped plate (Greiner Bio-One GmbH, #07-000-150), with each well containing 1 × 10^5^ cells. The plate was incubated for 30 min at 37 °C in a 5% CO_2_ atmosphere, followed by centrifugation at 500 × *g* for 3 min. Thereafter, 100 μL of a dilution series (10 μM, 2.5 μM, 625 nM, 156.3 nM, 39.1 nM, and 9.8 nM) of the different cy5-labeled compounds in IMDM complete was added to the individual wells containing cells. The cells were further incubated for 20 min at 37 °C in a 5% CO_2_ atmosphere. Following the incubation, the cells were centrifuged at 500 × *g* for 3 min, washed with PBS, and subjected to staining using the Zombie UV fixable viability kit (BioLegend, #423107) according to the manufacturer’s instructions. The stained cells were then incubated for 30 min at 37 °C with a 5% CO_2_ atmosphere. Subsequently, the cells were centrifuged, washed twice with 150 μL of PBS, and centrifuged again. Finally, the cells were resuspended in 200 μL of PBS, and the resulting cell suspension was analyzed using a BD LSR Fortessa Flow Cytometer (BD Biosciences) equipped with an HTS module. For data analysis, BD FACSDiva and FlowJo v10 Software were used. The gating strategy is presented in the Supporting Information (Figure S7). Three independent biological replicates were carried out. The mean ± SD of the median fluorescence intensity (MFI) values are presented. The fluorescence intensities were normalized according to UV/vis measurements of an aliquot of the aqueous stock solutions of each compound used for biological experiments in ethanol. The theoretical concentration of the measured solutions was 5 μM, and the absorption at 645 nm was used for the calculations. Ethanol was chosen as a solvent, as the formation of H-stacks of two or more cy5 groups in aqueous solutions of compounds 1a–1c, 2a, and 3a was observed. As interactions of the compounds with the cell membrane can be expected to prevent the formation of H-stacks, normalization to the UV/vis absorption of the compounds in aqueous solution was not feasible.

#### 2.4.5 Confocal Laser Scanning Microscopy

To gain further insights into the localization and uptake processes of the compounds within cells, live-cell imaging was carried out on the HEK293 cell line for selected compounds (1b and 1d, 2b and 2d, and 3b and 3d) labeled with cy5. HEK293 cells were washed with PBS, harvested, and counted. Subsequently, the desired cell quantity in IMDM complete was seeded into a 96-well glass-bottom plate (Cellvis, #P96-1.5H-N) and incubated for 48 h under standard cell culture conditions described above. Afterward, the cells were washed with PBS and treated with a staining solution comprising 100 μL of 2.5 μM of the selected labeled compounds and 2 μg mL^–1^ of Hoechst 34580 (Invitrogen, #H21486) as cell nuclei counterstain in phenol red-free IMDM (medium without FBS; Gibco, #21056023). The cells were then incubated for 20 min at 37 °C in a 5% CO_2_ atmosphere. After incubation, the solution was aspirated, the cells were washed with PBS, and 100 μL of phenol red-free IMDM were added. To capture the stained cells, along with appropriate controls, the Zeiss LSM 980 confocal microscope was utilized. The microscope was equipped with a water-immersion C-Apochromat 40*x*/1.2 W Corr objective and a gallium arsenide phosphide (GaAsP) photomultiplier tube (PMT) as the detector. The cells were imaged at 37 °C (at 30, 45, and 60 min post-incubation) with a 639 nm laser for cy5 and with a 405 nm laser for Hoechst 34580. All images were analyzed using ZEN 3.8 software (Carl Zeiss Microscopy).

The U251-MG cells were seeded in DMEM complete and subsequently incubated, as described above, with the respective compounds from each group (1b, 2b, and 3b) at both 37 and 4 °C. This was carried out in phenol red-free, high-glucose DMEM (medium without FBS; Gibco, #21063029), further referred to as phenol red-free DMEM. For pathway inhibitor studies, the U251-MG cells were respectively treated with specific inhibitors as follows: 50 μM LY294002 (InvivoGen, #tlrl-ly29) for 1 h, 200 μM monodansylcadaverine (MDC; Sigma-Aldrich, #30432) for 30 min, and 20 μM Pitstop 2 (Sigma-Aldrich, #SML1169) for 20 min. Subsequently, the cells were treated with the representative compound 1b, labeled with cy5, and with Hoechst 34580 for cell nuclei counterstain in phenol red-free DMEM, following the imaging procedure described above (captured at 15, 30, 45, and 60 min post-incubation). For colocalization studies, cells were stained with LysoTracker Green DND-26 (Invitrogen, #L7526) or MitoSpy Orange CMTMRos (Biolegend, #424803) for 30 min. They were then treated with representative compound 1b, labeled with cy5, and Hoechst 34580 for cell nuclei counterstain in phenol red-free DMEM, as described above. Imaging was performed at 37 °C using lasers with a wavelength of 405 nm for Hoechst 34580, 488 nm for LysoTracker, 561 nm for MitoSpy, and 639 nm for cy5.

#### 2.4.6 Statistical Analysis

Biological experiments were carried out in triplicate and are presented as the average value ± SD if not mentioned otherwise. The statistical significance of the differences was probed using one-way analysis of variance (ANOVA). Significance levels are either given as the p-value or marked within figures as follows: *: *p* < 0.05, **: *p* < 0.01, ***: *p* < 0.001, and ****: *p* < 0.0001.

## 3 Results and Discussion

### 3.1 Synthesis and Functionalization of the Oligopeptoids

**Scheme 1:**
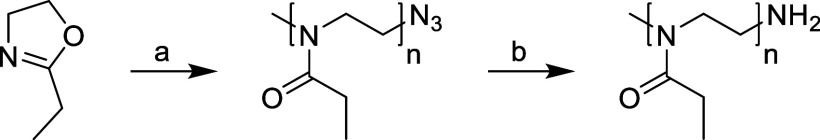
Polymerization of 2-Ethyloxazoline for the Generation of Polymer Fragments to Connect on the Solid Phase (a): MeTos, ACN, 100 °C, 4h, then NaN_3_ (5 equiv), 100 °C, 18 h; (b): Pd/C (10 wt %), Et_3_SiH, MeOH, rt, 1 h, or PPh_3_, THF, 0 °C to rt, 6 h, then H_2_O, rt, 18 h *n* ≈ 20.

To create exactly defined, branched polymers via solid-phase synthesis on a peptoid backbone, OEG, PEG, and PEtOx derivatives were used as fragments. The OEG- and PEG-based oligopeptoids were synthesized using commercially available PEG derivatives carrying a terminal primary amino group. Amine-terminated PEtOx fragments were synthesized via cationic ring-opening polymerization of 2-ethyloxazoline using methyl tosylate as the initiator. The polymerization was quenched with sodium azide, and the resulting azide end group was converted to an amine group in a postpolymerization modification reaction (Scheme 1).

Despite the fact that poly(2-methyloxazoline) (PMeOx) as the most hydrophilic poly-2-oxazoline polymer shows advantages compared to PEtOx, e.g., its derivatives adsorb less proteins from blood serum and show improved antifouling properties when used as surface coating,^61−63^ poly(2-ethyloxazoline)s were used as side chains for the generation of peptoids. 2-Ethyloxazoline has been proven easy to polymerize due to the low chain transfer rate in comparison to polymerizations of 2-methyloxazoline,^61,64^ and both polymers show similar properties when it comes to interactions with salts and also the potential to pass lipid bilayer membranes.^65^ Additionally, it would be possible to synthesize suitable PMeOx derivatives and connect them via solid-phase synthesis in upcoming studies.

**Scheme 2:**
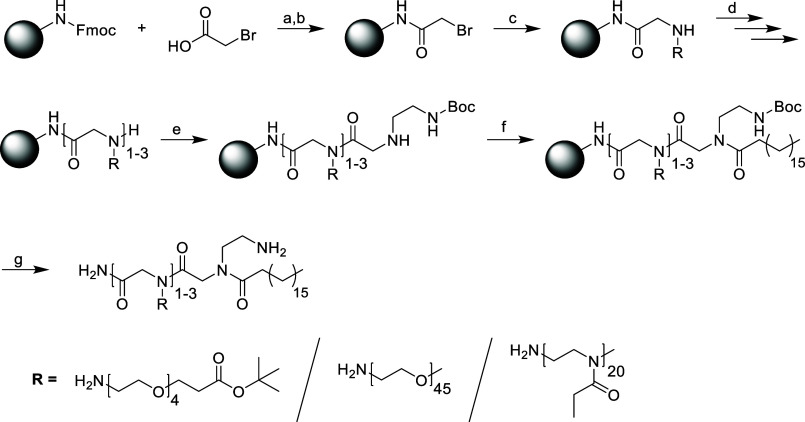
Connection of PEG or PEtOx Fragments on a Peptoid Backbone via Solid-Phase Synthesis (a): 20% piperidine in DMF, rt, 2 min, then 15 min; (b): bromoacetic acid, DIC, rt, 1 h; (c): OEG-NH_2_ or PEG-NH_2_ or PetOx-NH_2_-fragment, DMF, rt, 3 h–1 d; (d): steps b and c are repeated 0–2 times; (e): step b is repeated, then *N*-Boc-ethylenediamine, DMF, rt, 3 h; (f): stearic acid, DIC, DMF, 40 °C, 18 h; (g): TFA/H_2_O 95:5, rt, 1 h.

The peptoid backbone was grown from a rink amide resin using a two-step iterative approach. After the removal of the Fmoc protective group from the rink amide resin, acetylation of the free amino group was carried out using an excess of bromoacetic acid and DIC as a coupling agent in DMF. In the second step, an excess of the desired primary amine was added as a solution in DMF to form a secondary amine. Reaction times were chosen according to the molecular weight of the oligomeric or polymeric fragments; while the reaction was completed within a few hours for the OEG fragments (3 h in our case, specifically), reaction times of around 24 h were required for PEG and PEtOx fragments with a molecular weight of 2000 g mol^–1^. Further, replacing the amine solution with a freshly prepared one during the coupling process was helpful to avoid incomplete conversion in the latter case. The two steps were repeated until the desired number of OEG, PEG, or PEtOx building blocks were connected. After another acetylation reaction, a Boc-protected amino group was introduced in the displacement reaction using *N*-Boc-ethylenediamine. As a last step, the molecule was end-capped using stearic acid in a last acetylation reaction. Stearic acid does not contain a bromine group; therefore, no further growth of the peptoid backbone is possible afterwards. After completion of the synthesis, the product was cleaved from the resin using a mixture of TFA and water (95:5). The synthesis steps as well as the final products are shown in Scheme 2.

The generated structures show several features, making them suitable carrier molecules for drugs in polymer-drug conjugates. First of all, the exactly defined number and molecular weight of hydrophilic side chains determine the solubility properties of the molecules. If a hydrophobic end group is used, different structures (small micelles or molecularly dissolved, coiled chains) can be obtained in aqueous environments. Further, if only side chains with reactive end groups are used, one or several drug molecules can be attached to the carrier. Some membrane proteins occur in pairs or with a defined distance from each other; therefore, multifunctional drug carriers with a specific distance between the connected drugs may be of great interest.^15^ In our case, only the low-molecular-weight PEG chains carry a protected carboxylic acid as a reactive end group; to generate polymer-drug conjugates from the structures containing PEG or PEtOx with a molecular weight of 2000 g mol^–1^, the introduction of a reactive group instead of the terminal methyl group would be necessary. Nevertheless, the presented structures serve as model compounds to investigate the properties of these carriers in solution and toward lipid bilayer membranes.

**Scheme 3:**
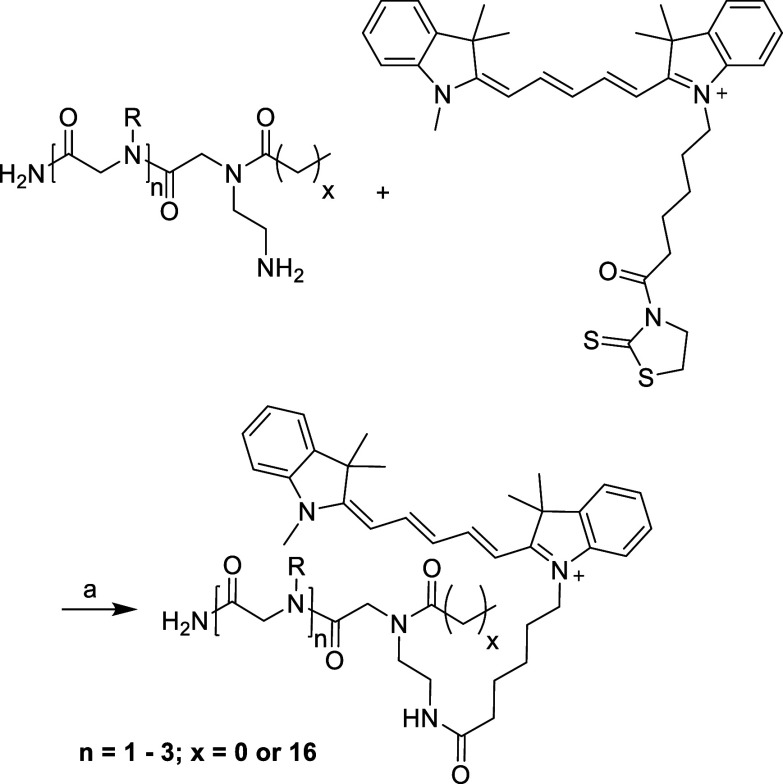
Attachment of a cy5-Dye to the Structures Obtained from Solid-Phase Synthesis (a): peptoid, cy5-2-mercaptothiazoline (cy5-TT, 3 equiv), diisopropylethylamine (DIPEA), DCM, rt, 18 h, dark.

The amino group is deprotected during the cleavage of the final product from the resin and can then be used for further functionalization of the molecule. In our case, a cy5-based fluorescent dye was introduced in this position to be able to track the molecule during biological studies (Scheme 3). The synthesis of the dye was realized in five steps from commercially available substances, as described in the Materials and Methods Section. The carboxylic acid of the dye was converted into a reactive derivative using 2-mercaptothiazoline (2-thiazoline-2-thiol, TT), which allows for quantitative coupling of the dye to the free amino group of the carriers (Scheme 3).

As a last point, we chose stearic acid as an end group for our systems to enable attachment to lipid bilayer membranes. This may increase the efficiency of polymer-drug conjugates when targeting membrane-bound proteins. Intercalation of the stearic acid into the cell membrane may localize the drug close to its target, thereby increasing its residence time. Additionally, reversible aggregation, or attachment to proteins, can prevent premature clearance of the carriers from the bloodstream via the kidneys. To investigate the influence of the stearyl anchor on lipid bilayer membranes in later studies, control samples with two PEG or PEtOx side chains and a nonhydrophobic acetyl end group were synthesized. The structures are depicted in Figure 1, and their chemical properties are described in Table 1.

**Table 1:** Chemical Description of Compounds 1a–1d, 2a–2d, and 3a–3d

compound	number of arms	end group	molecular weight or *M*_n_ (g mol^–1^, NMR)
**1a**	1	stearic acid	1154.6
**1b**	2	stearic acid	1459.9
**1c**	3	stearic acid	1765.2
**1d**	2	acetic acid	1235.5
**2a**	1	stearic acid	2900 (*M*_n_)
**2b**	2	stearic acid	5000 (*M*_n_)
**2c**	3	stearic acid	7100 (*M*_n_)
**2d**	2	acetic acid	4700 (*M*_n_)
**3a**	1	stearic acid	2900 (*M*_n_)
**3b**	2	stearic acid	5000 (*M*_n_)
**3c**	3	stearic acid	7100 (*M*_n_)
**3d**	2	acetic acid	4700 (*M*_n_)

After the synthesis, the products were purified using suitable Sephadex columns. ^1^H NMR measurements were carried out to confirm their structures (Figure 2A,C,E). HPLC measurements of structures 1a–1d (Figure 2B) and SEC measurements of structures 2a–2d and 3a–3d (Figure 2D,F) show a shift in elution volume according to the number of side chains on the peptoid backbone and further confirm the successful removal of excess dye. Structures 2a–2d show low dispersities (*D̵* = 1.03–1.20) and monomodal peaks in SEC traces, which proves that no significant amounts of side products with a lower number of PEG chains than targeted are present. In compounds 3a–3d, poly(2-oxazoline) chains (2000 g mol^–1^) that were not connected to the desired peptoid backbone were detected as a side product. The side product was removed from the samples containing stearic acid anchors by adsorbing the desired product on an Amberlite-XAD4 hydrophobic resin in water. The majority of the exclusively hydrophilic side product remains in the aqueous phase and can be discarded, while the desired peptoid can then be stripped from the resin using an organic solvent, such as THF. For compound 3d, removal of the side product as described was not possible. Nevertheless, as no free amine groups were detected in the side product that was separated from compounds 3a–3c, it can be assumed that the cy5 dye can only be attached to the desired product and not to the side product present in sample 3d. Therefore, the sample was used without the removal of the side product, and the amount of sample used in experiments was normalized according to UV/vis-absorption. The molecular weights of polymeric compounds were calculated from ^1^H NMR spectra using the stearyl or acetyl group as a reference. As solid-phase synthesis is optimized to obtain full conversion of the reactions carried out, large discrepancies from the expected molecular weight are not probable. The molecular weight of structures 1a–1d was additionally determined via MALDI-MS.

### 3.2 Solution Behavior of the Oligopeptoids

The number and molecular weight of the side chains attached to the peptoid backbone were expected to determine the behavior of the structures in solution. The investigation of the latter via DLS led to results with high uncertainty due to the low intensity of the scattered light in most samples, as the compounds do not form solution structures with a large and dense hydrophobic core. The chromophore of the cy5-dye can absorb light from the probe laser; hence, measurements had to be carried out on the substances before attachment of the fluorophore. For compounds 1a–1d, the attached fluorophore may change the solubility in water due to its high molecular weight compared to the oligopeptoid. Therefore, the results obtained from DLS were only used to estimate if larger aggregates are present at higher concentrations of the compounds. These particles may be loose aggregates of smaller micelles that are not detected due to the lower intensity of light scattered by small structures.^66^ The hydrodynamic radius of these smaller particles was determined by FCS.^67,68^ A mixture of labeled and unlabeled peptoid stock solutions in water (1 mg mL^–1^) was used in each sample to adjust the fluorescence intensity of the samples without varying the peptoid concentration from sample to sample. The samples were analyzed directly after mixing, in case thermal equilibration and the resulting formation of mixed aggregates of labeled and unlabeled compounds would change the nature of the present aggregates. Block copolymers with larger hydrophobic segments often exhibit kinetic trapping of aggregates,^69^ surfactants, and surfactant-like structures, as the compounds presented here normally exist in an equilibrium state. Aggregates as well as freely dissolved molecules are present in solution and are able to form mixed aggregates after equilibration of the system.^70^ The diffusion coefficients of the solution structures as well as the radii determined from the latter via the Stokes–Einstein equation are listed in Table 2. The obtained values lead to the conclusion that aggregation of the compounds with hydrophobic anchor into small micelles with a hydrodynamic radius of 5–10 nm takes place, while compounds without hydrophobic anchor or with very large hydrophilic fractions are dissolved as single molecules. The hydrodynamic radius of these dissolved macromolecules ranges from 2–3 nm. A larger hydrophilic portion in the compounds led to small supramolecular assemblies (loose aggregates of a smaller number of molecules and unimers), which is most likely caused by steric reasons (lower critical packing parameter).^71,72^ For example, the hydrodynamic radius of compounds 2c and 3c, determined by FCS measurements, was 3.4 ± 0.2 and 5.6 ± 0.7 nm, respectively, while compounds 1a and 2a exhibit a hydrodynamic radius of more than 10 nm.

**Table 2:** Aggregation Behavior of the Structures in Aqueous Solution at High Concentrations: Diffusion Coefficients and Hydrodynamic Radii Determined via Fluorescence Correlation Spectroscopy and the Presence of Larger Particles in DLS Measurements

compound	(FCS, 1 mg mL^–1^, micropure water)			(DLS, 1 mg mL^–1^, micropure water)	(DLS, 1 mg mL^–1^, PBS, pH = 7.4)
	*D*_T_/μm^2^ s^–1^	*R*_H_/nm	pH value	particles > 50 nm	zeta potential/mV
1a	20.0 ± 2.6	12.4 ± 1.9	4.66	√	–29.52 ± 0.94
1b	11.7 ± 2.4	21.3 ± 3.9	3.72	√	–19.32 ± 0.126
1c	17.6 ± 4.5	7.6 ± 1.7	5.80		–20.86 ± 0.68
1d	134.3 ± 34.3	1.9 ± 0.6	5.83		–11.72 ± 0.94
2a	24 ± 3.2	10.2 ± 1.4	4.28	√	–1.16 ± 0.25
2b	27.0 ± 1.6	8.8 ± 0.7	5.5		–0.30 ± 1.38
2c	72.3 ± 5.1	3.4 ± 0.2	5.01		–13.00 ± 1.18
2d	133.1 ± 4.2	1.9 ± 0.5	5.21		–14.14 ± 0.96
3a	37.2 ± 3.0	6.7 ± 0.9	7.05	√	–4.38 ± 0.56
3b	35.9 ± 2.7	7.3 ± 2.3	6.37	√	–4.58 ± 0.41
3c	44.0 ± 2.2	5.6 ± 0.7	6.62		–6.38 ± 0.43
3d	87.2 ± 16.6	2.8 ± 0.5	6.01		–6.27 ± 0.77
cy5-COOH	230.0 ± 37.0	1.1 ± 0.4			

It has to be noted that the solutions of compounds 1a–1d and 2a–2d in micropure water exhibited a slightly lower pH value than the solutions of compounds 3a–3d (pH ≈ 4.5–5.5 for compounds 1a–d and 2a–2d in comparison to pH ≈ 6–7 for compounds 3a–3d). The pH value can influence the aggregation behavior of the compounds, especially for compounds 1a–1d, which can be partially deprotonated in solution, leaving behind a surfactant with one or multiple negative charges in the hydrophilic compartment. This may increase the solubility of the compound as a whole in comparison to the uncharged species 2a–2d and 3a–3d, despite the fact that the hydrophilic chains incorporated are shorter. The particles may as well be less stable and exhibit a lower aggregation number than particles with uncharged side chains of the same length due to the electrostatic repulsion of the negatively charged groups.

Zeta potential measurements in diluted PBS buffer (pH = 7.4) prove partial deprotonation as compounds 1a–1c exhibit a negative Zeta potential in between −30 and −20 mV, while particles from compounds 2a–2c and 3a–3c can be classified as neutral (Zeta potential of around ±0 and −4, respectively). The measured Zeta potential is given in Table 2. It has to be noted that for compounds with low scattering intensity (e.g., compounds 2c and 3c), i.e., compounds that do only form very loose aggregates or do not form aggregates at all (compounds 1d, 2d, and 3d), the determined values for the Zeta potential are less reliable.

**Figure 3:**
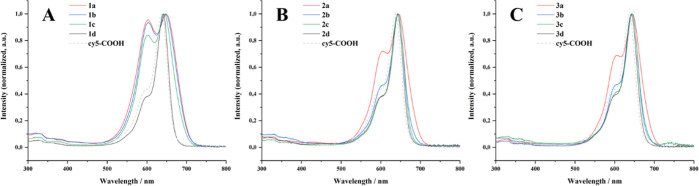
UV/vis absorption spectra of compounds 1a–1d (A), 2a–2d (B), and 3a–3d (C), compared to the free dye in solution (cy5-COOH was used for the measurements, as the direct precursor, cy5-TT, decomposes to cy5-COOH in water). The concentration of the compounds was set to 10 μg mL^–1^ to obtain reasonable absorbance values.

Further, UV/vis absorption spectra of the compounds in aqueous solution, as well as fluorescence excitation and emission spectra, are not only valuable for choosing suitable experimental settings in biological experiments; they also provide additional information about the aggregation behavior of the substances.^73,74^ It is known that cyanine dyes are able to form so-called H-stacks of two or more molecules, which shifts their absorption maximum toward lower wavelengths (hypsochromic shift).^75^Figure 3 shows that the UV/vis spectra of more hydrophobic compounds (1a–1c, 2a, and 3a) exhibit a second absorption maximum, or a pronounced shoulder, at 600–605 nm, while the absorption maximum of the nonaggregate dye ranges from 640–650 nm. As the dye is covalently bound to the compounds and neighbors the hydrophobic anchor, aggregation of the compounds leads to the proximity of the dye molecules and to the formation of, e.g., cy5 dimers or larger stacks.

**Figure 4:**
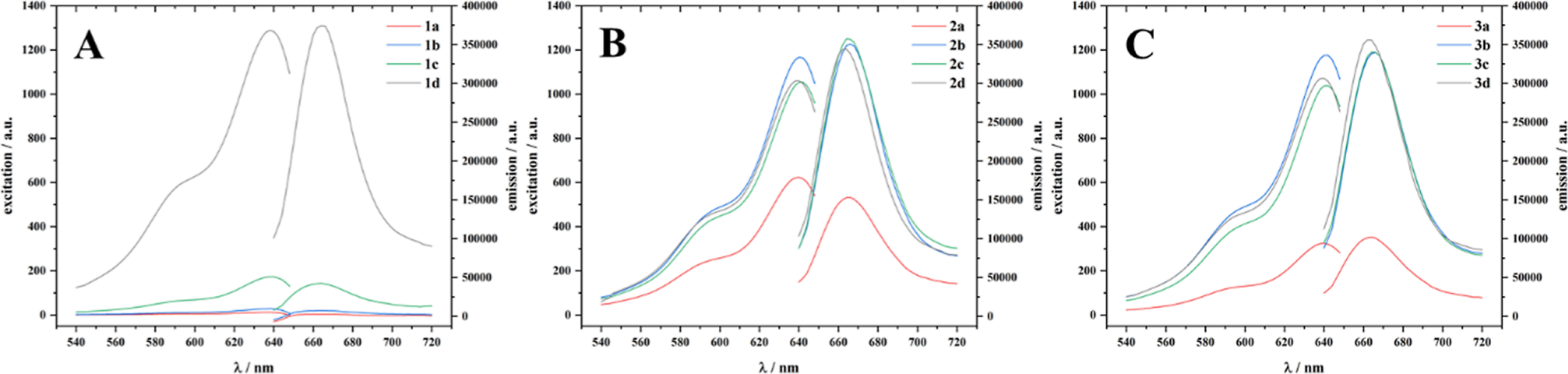
Fluorescence excitation and emission maps for compounds 1a–1d (A), 2a–2d (B), and 3a–3d (C). Excitation maps were measured at a detection wavelength of 660 nm, while emission spectra were measured at an excitation wavelength of 638 nm. The concentration of the compounds was set to 10 μg mL^–1^ to obtain reasonable absorbance and emission values. A magnified map for compounds 1a–1c can be found in the Supporting Information (Figure S2).

Fluorescence excitation–emission maps further demonstrate that aggregation of the cy5 dyes quenches the fluorescence of the compounds (Figure 4). The effect is again visible for compounds 1a–1c, 2a, and 3a, which show decreased fluorescence emission compared to the substances with higher hydrophilicity. It can be assumed that the fluorescence signal arises partially from aggregated compounds, if present, due to incomplete quenching. Nevertheless, the higher share of the detected fluorescence arises from polymer–dye conjugates that are freely dissolved due to either the low tendency of the compound to form aggregates or the equilibrium between aggregated and freely dissolved structures.

Foregoing measurements only revealed the presence or absence of aggregates at one concentration. These concentrations were comparably high (1 mg mL^–1^), while UV/vis, fluorescence, and FCS measurements need to be optimized to obtain suitable absorption or emission ranges. To investigate the behavior of the compound in solution over a larger concentration range, the critical micelle concentration (CMC) was determined for all compounds to investigate whether the presence of the stearyl group induces micellization at a specific concentration. The CMC is an important parameter to keep in mind when studying the attachment of the compounds to membranes, as it determines the concentration of stearyl groups accessible in solution for interactions with lipid bilayers. Above CMC, different amounts of the hydrophobic anchor are shielded within the core of a micelle, depending on the CMC value of the respective compound. Interactions of the compounds with membranes would still be expected, but the rate-determining step in the attachment process may be the disassembly of micelles during the restoration of the micelle–unimer equilibrium state. Therefore, it seemed beneficial to choose concentrations below the CMC for follow-up experiments, if possible.

For the CMC determination, a solution of pyrene in PBS (33 nM) was mixed with solutions of unlabeled oligopeptoids in PBS at different concentrations. While pyrene is fluorescent in hydrophobic environments, e.g., in the core of a micelle, its fluorescence is strongly quenched in aqueous solution. The CMC values, determined using the change in fluorescence intensity of the first and third vibronic peaks in the fluorescence emission spectrum, are presented in Table 2.^76^ Fluorescence-concentration plots can be found in the Supporting Information (Figures S3–S5). The ratio of the third to the first vibronic peak in the fluorescence spectra (*I*_3_/*I*_1_) was determined for all measurements as well, as it is dependent on the chemical environment of the pyrene molecule and can therefore be used for CMC determination as well.^77^ In this case, using the absolute fluorescence intensities of the peaks proved to be a more reliable method due to the large fluctuation of the *I*_3_/*I*_1_ value. As expected, the CMC values were higher for compounds with a larger hydrophilic share. No micellization was detected for compounds without a hydrophobic anchor within the investigated concentration range of 0.1 μM to 1 mM (2 mM in the case of 1c).

Summarizing the results presented in Tables 2 and 3, it seems probable that compounds with a hydrophobic anchor and one or two hydrophilic side chains form small micelles that exist in an equilibrium state with single chains in solution. For smaller compounds (1a and 1b), or compounds that can be regarded as linear polymers (1a, 2a, and 3a), regular micelles with a core consisting of the stearyl groups are formed, which leads to the proximity of the cy5 dye in labeled compounds and therefore to the formation of H-stacks and fluorescence quenching. For compounds 2b and 3b, the core region may be less defined due to steric reasons, thereby preventing the formation of H-stacks within the micelles. Compounds 1c and 1d, 2c and 2d, and 3c and 3d exist as unimers in solution up to a comparably high concentration. Compounds 2c and 3c may form random coils shielding the stearyl anchor and the cy5 dye in their centers, thereby preventing the formation of H-stacks, while for compound 1c, aggregation of a small number of molecules is still possible due to its lower molecular weight. Compounds 1d, 2d, and 3d are not expected to form micelles or even dimers due to the lack of a hydrophobic unit.

**Table 3:** Behavior of the Compounds in Solution at Low Concentrations: CMCs and the Presence of H-Stacks (Visible in UV/Vis) That Lead to Fluorescence Quenching

compound	CMC (μM)	H-stacks/fluorescence quenching
1a	1.5 ± 0.1	√
1b	36.8 ± 1.3	√
1c	454 ± 26	√
1d	>1000	
2a	29.7 ± 1.9	√
2b	30.4 ± 5.5	
2c	339 ± 14	
2d	>1000	
3a	34.1 ± 1.9	√
3b	43.4 ± 1.6	
3c	320 ± 10	
3d	>1000	
cy5-COOH		

Further investigations were carried out at concentrations below ≈30 μM, as micelle formation at these concentrations would only be expected for compound 1a. Fluorescence quenching may be observed for compound 1a during these experiments or even for other compounds forming dimers.

### 3.3 Interaction of Oligopeptoids and Lipid Bilayer Membranes

**Scheme 4:**
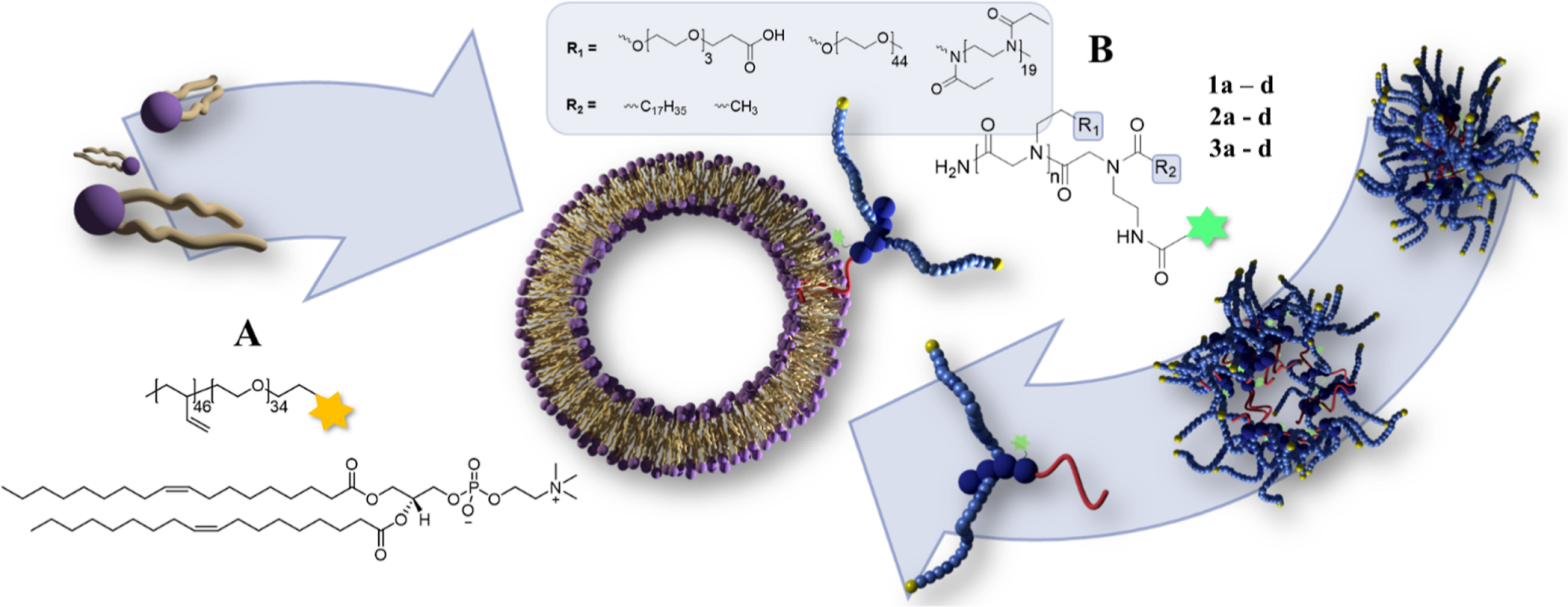
Liposomes were Generated From DOPC and poly(ethylene oxide)-*b*-1,2-poly(butadiene) (PEO-*b*-PBD) (A), Purified, and Incubated with Oligopeptoids (B) to Investigate Their Ability to Bind to Lipid Bilayer Membranes

**Figure 5:**
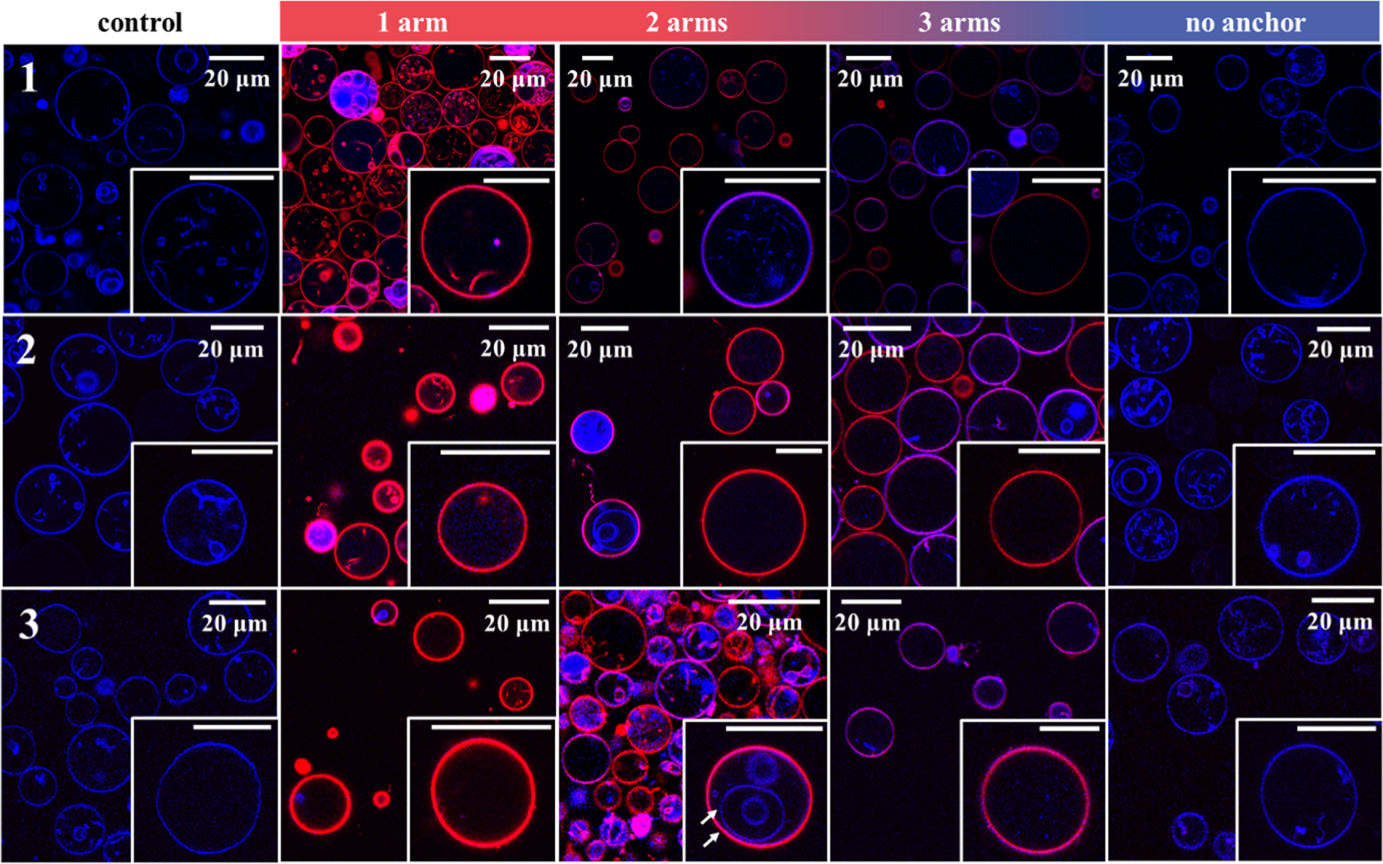
CLSM images of liposomes incubated with oligopeptoids 1a–1d, 2a–2d, and 3a–3d (see numbers on the left) for 3 h. The control samples show liposomes of the same batch without any oligopeptoid added. Scale bars in magnification: 20 μm; blue channel: excitation 405 nm, emission 440–480 nm, atto390; red channel: excitation 635 nm, emission 700–770 nm, cy5.

While *in vitro* studies using cultivated cell lines can provide a variety of information about the mechanisms that help synthetic substances cross cell membranes, passive interactions between lipid bilayer membranes and these substances can be investigated using liposomes, which can act as simple cell membrane models without active transmembrane transport mechanisms.^51^ Liposomes can be generated from phospholipids by extrusion^52^ or electroformation,^78^ yielding uni- or multilamellar vesicles of different sizes. As we were interested in the ability of our compounds to attach to, or intercalate into, cell membranes, studying the interactions between liposomes and the oligopeptoids presented in this manuscript proved to be helpful not only to obtain first information about oligopeptoid–membrane interactions, but also to distinguish between passive and active interactions in later cell studies. The liposomes used for our studies were generated from DOPC and poly(ethylene oxide)-*b*-1,2-poly(butadiene) (PEO-*b*-PBD, 5 mol % labeled with atto390) (5:1). The block copolymer is used as an additive to both stabilize and fluorescently label the lipid bilayer membranes (see Scheme 4). A thin film of these compounds was used to generate giant vesicles in sucrose solution via electroformation. The liposomes were purified by adding a glucose solution to the crude liposome solution, which induced sedimentation of the liposomes, while nonassembled phospholipids and block copolymers stayed evenly distributed within the solution. The purified liposomes were carefully taken from the bottom of the vessel with an Eppendorf pipet and incubated with the oligopeptoids at a concentration of 10 μmol for 3 h. Excess peptoid was removed by another washing step with glucose solution before the samples were investigated via confocal CLSM (Figure 5).

The images clearly demonstrate that the ability of the oligopeptoids to attach to the lipid bilayer membranes of the liposomes depends on the ratio of the number of hydrophilic side chains to the hydrophobic anchor. For all three compound groups, the strongest binding was observed for compounds with only one hydrophilic arm and a hydrophobic anchor, followed by compounds with two and three arms and the hydrophobic anchor, respectively. Compounds with acetyl instead of stearyl end groups did not show any significant binding to the membranes. Further, the images reveal that without any active internalization processes, the compounds do not cross the liposome membranes in most cases, as no increase in the fluorescence intensity was detected for the inner hydrophilic compartments of the liposomes. It is also visible that for multilamellar vesicles, only the outer membrane is stained by the oligopeptoids in most cases (see, e.g., image of liposomes stained with compound 2b, white arrows in magnification). Exceptions were found for compounds 1a and 2a, where staining of membrane fragments encapsulated within liposomes was observed. Although all images were recorded using the same settings in CLSM, differences in fluorescence intensity can mainly be compared within one row in Figure 5, as the samples in different rows were prepared from different batches of liposomes that may vary in exact liposome concentration. Therefore, as samples 1a–1d cannot directly be compared to their respective polymeric, uncharged counterparts, the influence of the surface charge on the attachment to the liposomes cannot be quantified here. Nevertheless, it is obvious that the electrostatic repulsion which may occur in between the liposome surface and particles formed from compounds 1a–1d does not prevent the interaction of the peptoids and the membrane, probably due to the equilibrium of aggregated and free oligopeptoids in solution and the sufficient distance of the charged groups to the charged lipid head groups in the case of attachment of the compound.

**Figure 6:**
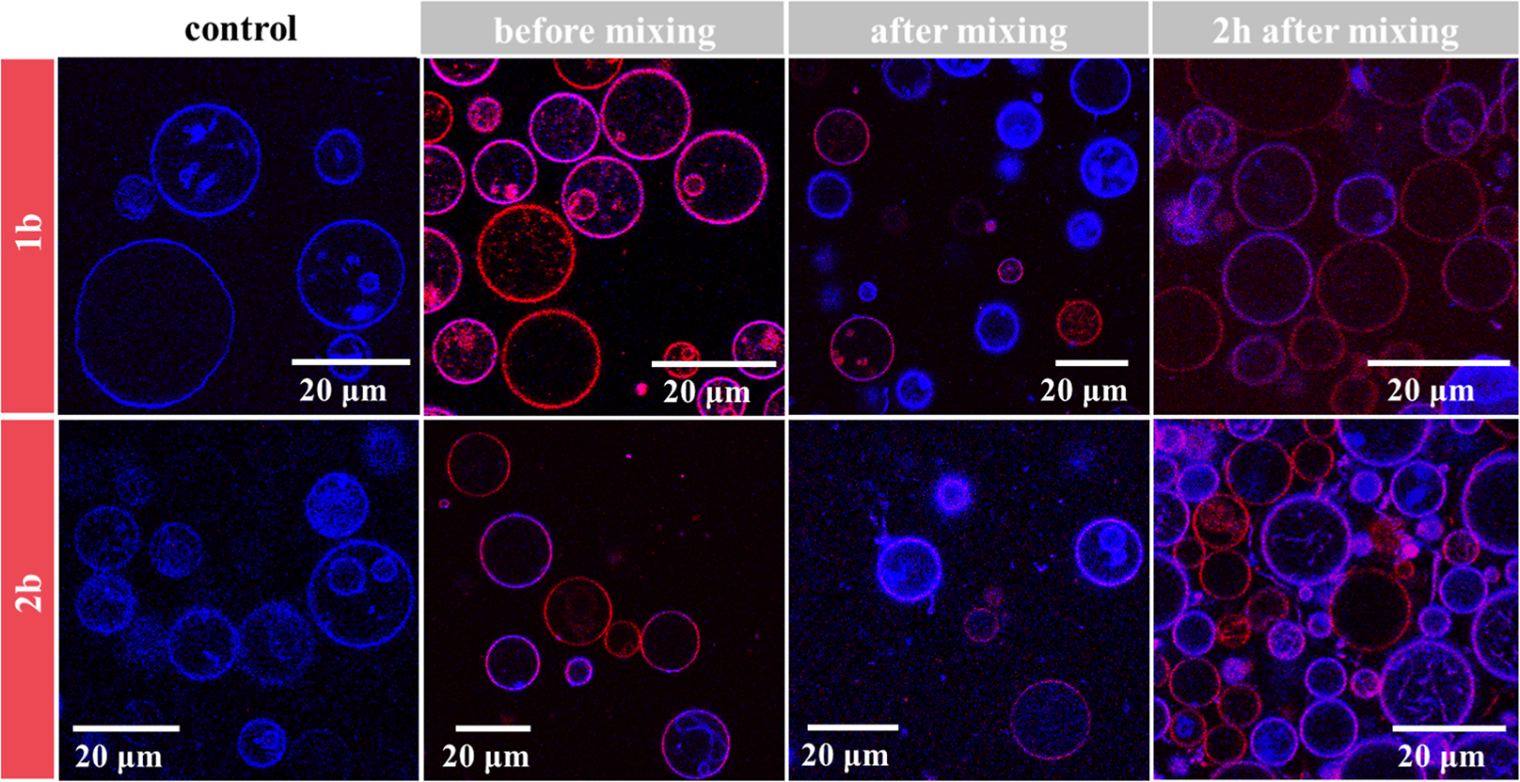
CLSM images of liposomes incubated with compounds 1b and 2b for 3 h, respectively. Nonincubated liposomes (control) were added, and images were taken directly after mixing both solutions and 2 h after mixing both solutions. Blue channel: excitation 405 nm, emission 440–480 nm, atto390; red channel: excitation 635 nm, emission 700–770 nm, cy5.

Membrane interactions can support uptake of compounds into cells, yet it is necessary that they are reversible to prevent permanent localization of a compound that is supposed to be internalized on the cell membrane or permanent blocking of cell surface receptors. Additional experiments to probe the reversibility of the binding process of the peptoids to the liposomes were carried out. First, a batch of liposomes was incubated with selected peptoids (1b and 2b), as described before. Then, an equal volume of a solution of incubated and nonincubated liposomes was mixed. CLSM studies of liposomes incubated with compound 1b revealed that labeled and unlabeled liposomes can be observed directly after mixing the two solutions, while at later time points, all liposomes found were evenly labeled. This suggests that the peptoids were able to detach from the liposome surface and bind to the freshly added, nonincubated liposomes. In the case of liposomes incubated with compound 2b, this process seems to be faster due to the lower hydrophobicity of the compound, as all liposomes exhibited cy5 fluorescence to some extent directly after mixing (Figure 6).

### 3.4 Cell Interaction Studies

While the preliminary experiments carried out on liposomes demonstrated the ability of the compounds to attach to the artificially prepared membranes, *in vitro* experiments with cells gave insights into the processes that may lead to the attachment and further to the uptake of the compounds into the cells. We studied the interactions of all compounds with HEK293 cells or U251-MG cells as model cell lines using flow cytometry and CLSM. While HEK293 cells were used as the primary cellular model for the basic interaction of given compounds with cell membranes, CLSM experiments that required extensive washing procedures were carried out on U251-MG cells due to their enhanced resistance towards detachment. Cytotoxicity and exemplary CLSM uptake studies were carried out for both cell lines; no significant differences were observed. The cells were cultivated in IMDM or DMEM complete at 37 °C in a 5% CO_2_ atmosphere in an incubator. All incubation experiments were carried out within a concentration range of 10 μM to 10 nM for a specified time with the respective compounds in IMDM or DMEM.

**Figure 7:**
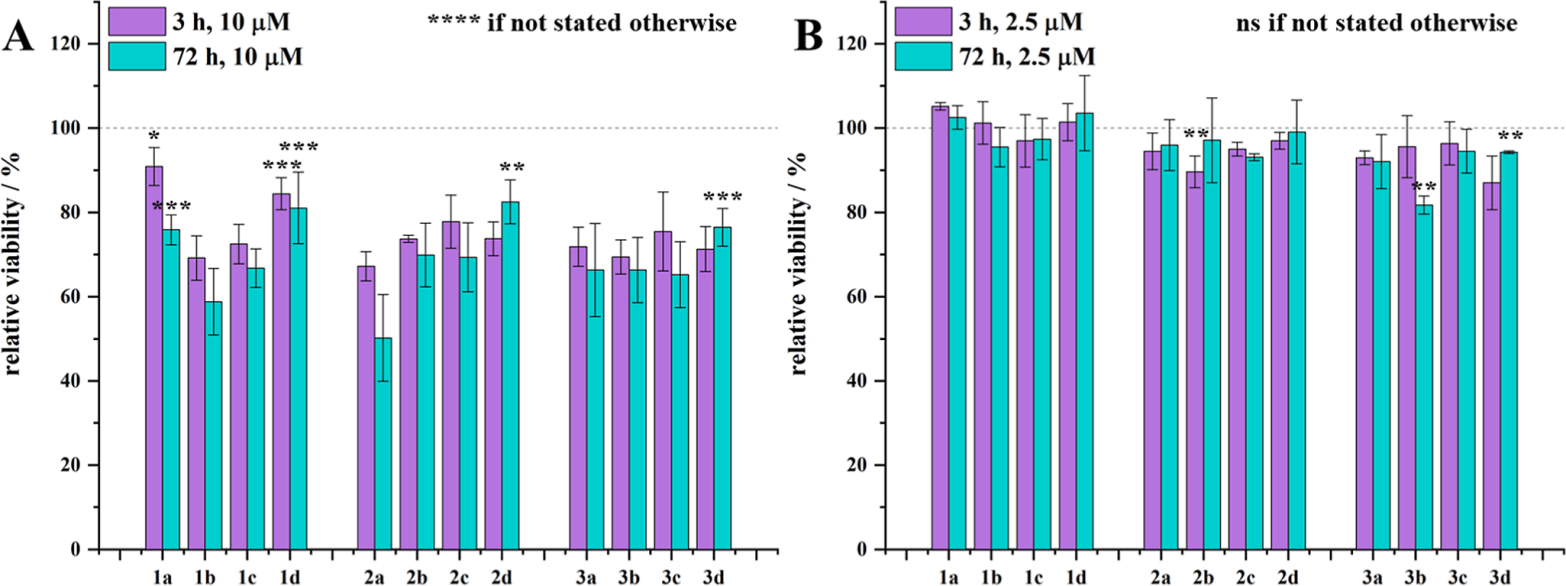
Relative metabolic activity of the HEK293 cells, normalized to the respective untreated control (3 or 72 h) in each assay, as determined by probing the ATP production of the cells via a luminescent-based assay. At compound concentrations of 10 μM, a significant decrease of metabolic activity was detected (A, *p* < 0.05 to 0.0001), while no significant changes were observed for compound concentrations of 2.5 μM for most samples after 3 and 72 h, respectively (B). Error bars represent ± SD (*n* = 3). Statistical significance was probed via one-way ANOVA, as described in Section 2.4.6. As described later, the concentration of 1a was lower due to the low solubility of the compound in aqueous environments (2 and 0.5 μM, respectively).

As a preliminary cell viability study using the luminescent-based assay for ATP measurement revealed that the cells exhibited decreased metabolic activity upon exposure to 10 μM of the compounds (average relative viability of 75% after 3 h and 70% after 72 h for HEK293 cells and 77% after 72 h for U251-MG cells), detailed interpretation of the cell–compound interactions was carried out for a concentration of 2.5 μM. At this concentration, no significant decrease of the metabolic activity of the cells was determined for up to 72 h (metabolic activity on average > 95% after 72 h for HEK293 cells, Figure 7, and >99% for U251-MG cells, Figure S6). The metabolic activity for the whole concentration range that was investigated is shown in the Supporting Information (Figure S7).

**Figure 8:**
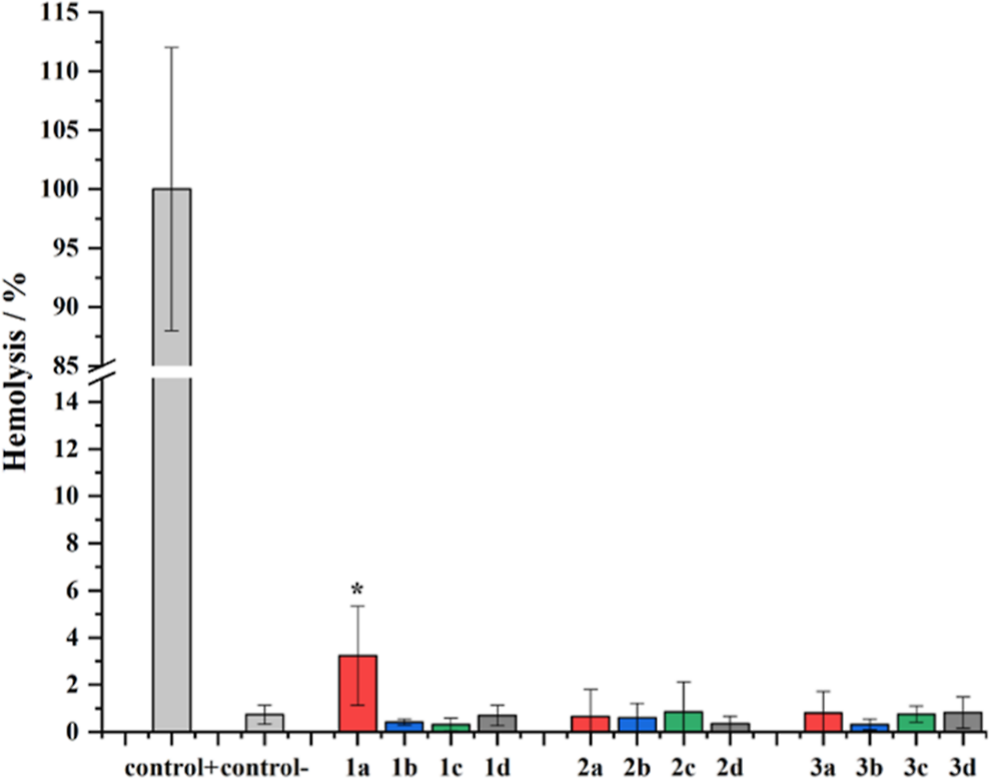
Hemolysis of human RBCs caused by all investigated compounds at a concentration of 10 μM. control+: 1% Triton X-100; control-: PBS. Compound 1a showed low hemolytic activity (*p* < 0.05). No significant differences were found comparing the negative control and the other samples. Error bars represent ± SD (*n* = 3). Statistical significance was probed via one-way ANOVA, as described in Section 2.4.6.

Amphiphilic substances, like the investigated compounds with a hydrophilic anchor, can act as surfactants and therefore not only attach to but also damage cell membranes. As cell membrane lysis is not desired when staining cell membranes or targeting membrane-bound proteins, it had to be excluded that the decrease in metabolic activity of the cells was caused by cell membrane disruption. Therefore, the hemolytic activity of the compounds was probed on human RBCs. In the case of cell membrane disruption of the RBCs, hemoglobin is able to exit the cells and can then be detected spectrophotometrically. A concentration range of 0.156 to 40 μM was investigated to cover concentrations with and without detected effects on cell viability in the luminescent-based assay. No significant hemolytic activity was detected for the concentration range investigated in toxicity and cell interaction studies except for compound 1a (Figure 8); therefore, cell membrane disruption or lysis is most likely not the cause for the decreased cell viability after incubation with 10 μM of the compounds. Additionally, compound 2a showed low hemolytic activity at higher concentrations (40 μM, Figure S8). This may be attributed to the increased hydrophobicity of compounds 1a and 2a, which leads to an increased tendency to interact with lipid bilayer membranes.^54^ This finding explains the fact that small amounts of fluorescent oligopeptoid were found inside of liposomes in the liposome incubation experiment, as these compounds may pass the cell membrane by locally and partially disrupting the lipid bilayer. The same effect can cause the leaking of hemoglobin from RBCs during the hemolysis experiments without completely disintegrating the cell membrane.

As no correlation between the decrease of metabolic activity and the structural properties of the compounds was determined and no significant cell membrane lysis occurred at the investigated concentrations, the compounds may be used for drug conjugation at moderate concentrations.

As a next step, flow cytometry experiments were carried out on HEK293 cells incubated with all compounds in a concentration range of 0.01–10 μM for 20 min. According to the ^1^H NMR spectra of the compounds, the degree of functionalization with cy5 was quantitative in most samples. Nevertheless, a slightly reduced degree of functionalization as well as the presence of water in the sample during weighing may lead to a certain error in detected fluorescence intensities in flow cytometry experiments. Therefore, an aliquot of the stock solutions of the compounds used for biological experiments was diluted in ethanol to a concentration of 5 μM, and the measured fluorescence intensities were used to correct the obtained results (Table S1). Ethanol was used as a solvent to prevent the formation of H-stacks.

**Figure 9:**
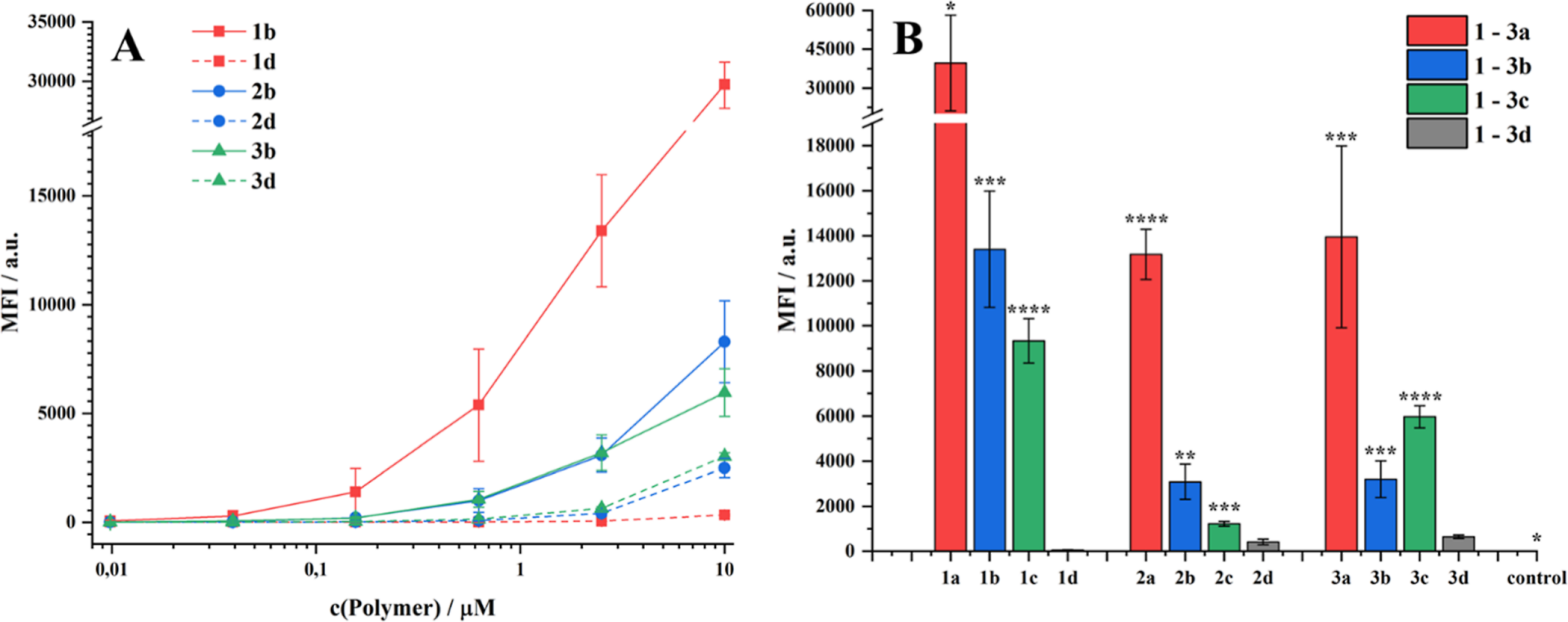
Increase of MFI with increasing concentration for selected samples (A) and comparison of binding affinity of all samples at 2.5 μM (B, corrected from 0.5 μM for compound 1a). Significance levels are displayed for each sample with hydrophobic anchor (1a–1c, 2a–2c, and 3a–3c) in comparison to the corresponding sample without hydrophobic anchor (1d, 2d, and 3d), and the lowest determined significance level of the control sample (untreated cells) in comparison to all other samples. Error bars represent ± SD (*n* = 3). Statistical significance was probed via one-way ANOVA, as described in Section 2.4.6.

An increase of the fluorescence signal of the cells with increasing concentrations of the compounds was observed, and no saturation effect was detected (Figure 9A). The fluorescence intensities of cells incubated with 2.5 μM of the compounds were utilized to compare the samples. This approach excluded the potential influence of the decreased metabolic activity observed in the cell viability assay when using 10 μM of the compounds. The studies revealed that the interactions of the compounds with HEK293 cells followed the same pattern as the attachment of the structures to lipid bilayer membranes that was demonstrated using liposomes (Figure 9B). Compounds with a higher number or molecular weight of the hydrophilic side chains were interacting with the cells at a lower rate compared to their more hydrophobic counterparts. The difference between polymeric compounds with only one hydrophilic arm and compounds with two or three hydrophilic arms was more pronounced than the difference between the latter (*p* < 0.005 vs *p* < 0.05 for compounds 2a–2c, and *p* < 0.05 vs no significant difference for compounds 3a–3c). The partially negatively charged hydrophilic compartment of compounds 1a–1c, or the negatively charged surface of particles formed from these compounds, may hamper interactions with the cell membrane. Nevertheless, this effect was not as pronounced as the differences caused by the molecular weight of the hydrophilic block in comparison to the hydrophobic anchor. Surprisingly, the fluorescence signal of the cells incubated with compound 1a seemed to be significantly lower than that of the cells incubated with compound 1b and not substantially higher than that of compound 1c. This can be attributed to the low solubility of compound 1a in water. According to UV/vis measurements, only 20% of the expected amount of labeled compound (0.5 μM instead of 2.5 μM) was properly solubilized in the stock solution used for the biological experiments. A Grubb’s test on the results of the UV/vis absorption measurements identified the absorption of compound 1a as an outlier. Therefore, despite the correction of the MFI according to the results from UV/vis spectroscopy, the obtained values for compound 1a may not be compared to the other flow cytometry results, as the conditions during incubation (e.g., concentration) were significantly different to the ones used for the other samples. Further, it may be possible that the partial micellization of the compound led to a lower binding affinity of the compound (binding affinity in this case refers to the nonspecific binding or internalization of the compounds to or into the cells). Micelles formed from compounds 1a–1c would exhibit a negative surface charge and therefore be repelled from the cell surface, which exhibits a negative charge as well.^79,80^ For a molecularly dissolved compound 1a, the assumed binding affinity would be higher than for compounds 1b and 1c, respectively. The difference between 1a and 1b is not statistically significant with *p* = 0.054 due to the high SD in sample 1a, while the difference between 1a and 1d is statistically significant with *p* < 0.05.

For all three compound groups, it can be stated that the affinity of the substances containing a hydrophobic anchor toward the HEK293 cells was significantly higher than for compounds with an acetyl end group (*p* < 0.05 for compound 1a, *p* < 0.005 for all other compounds). Interestingly, compounds 1d, 2d, and 3d did show low binding affinity in the conducted experiments compared to the control (*p* < 0.05), while this was not the case for the incubation of liposomes with the same substances. It is further visible that the median fluorescence intensity of cells incubated with compound 1d is significantly lower than that of compounds 2d and 3d, which can be attributed to its structure. The partially negatively charged carboxylic acid groups are repelled from the cell surface, which exhibits a negative charge as well, as already mentioned.^79,80^ As structure 1d does not contain a hydrophobic anchor, there are no attractive interactions between the substance and the cell surface. On the other hand, both higher molecular weight PEG and PEtOx do not exhibit a negative charge and may therefore show a low but notable affinity to the cell membrane, e.g., due to interactions of the moderately hydrophobic cy5 dye with the cells.

**Figure 10:**
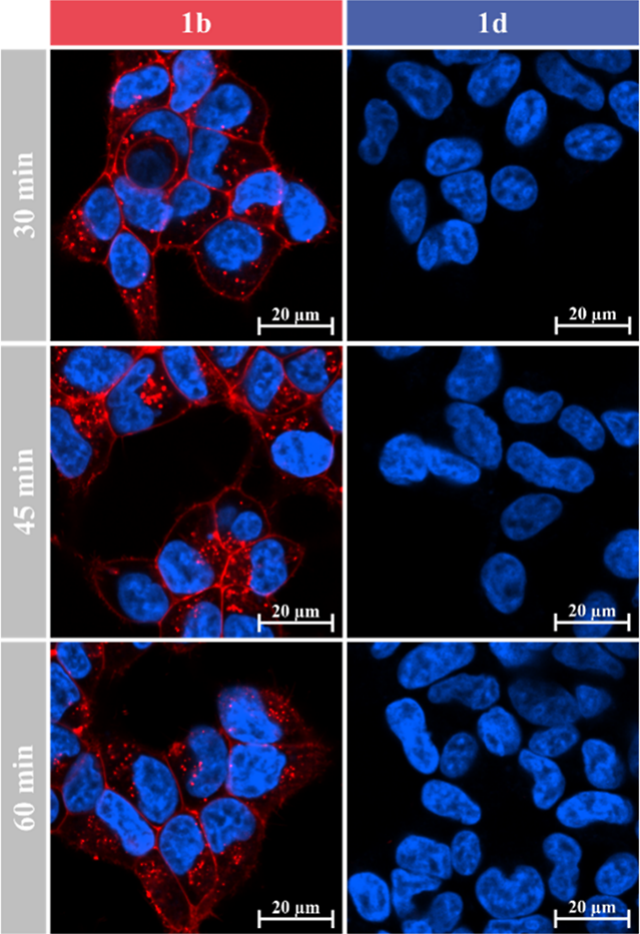
Live-cell CLSM images of HEK293 cells treated with compounds 1b (with hydrophobic anchor) and 1d (without hydrophobic anchor), recorded at different times after a 20 min incubation of the cells with the respective compounds. Cell nuclei were stained with Hoechst 34580. Blue channel: Hoechst 34580, laser wavelength 405 nm; red channel: cy5, laser wavelength 639 nm. Laser power for cy5 was set to 3%.

The assumption that the substances attach to cell membranes due to their hydrophobic anchor was probed by CLSM of the incubated HEK293 cells, recorded at different post-incubation times. For CLSM, the cultivated cells were incubated with the oligopeptoid compounds (2.5 μM) and Hoechst 34580 (2 μg mL^–1^) to counterstain the cell nuclei for 20 min in phenol red-free IMDM. For each set of compounds, the oligopeptoids with two side chains, with and without a stearyl end group, were investigated and compared (Figures 10 and S10 and S11). Microscopy pictures revealed that compounds with a hydrophobic anchor were attached to the cell membrane after 30 min. Furthermore, the uptake of the compounds into the cells was observable. As the fluorescence signal was not evenly distributed within the cell but rather clustered in distinct spots, uptake of the compounds via the formation of vesicles from the cell membrane (endosomes) and further transport of the substances encapsulated in membrane vesicles (e.g., exosomes or lysosomes) seems probable.

The fluorescently labeled polymer seems to pass the cell membrane in cell studies but did not pass the membrane of liposomes; therefore, it seems likely that internalization of the compounds occurs via active processes, e.g., clathrin- or caveolin-mediated transport.^81^ On the other hand, recent studies demonstrated that negatively charged, cy5-labeled polymers were able to passively diffuse through cell membranes and additionally target mitochondria.^82−84^

**Figure 11:**
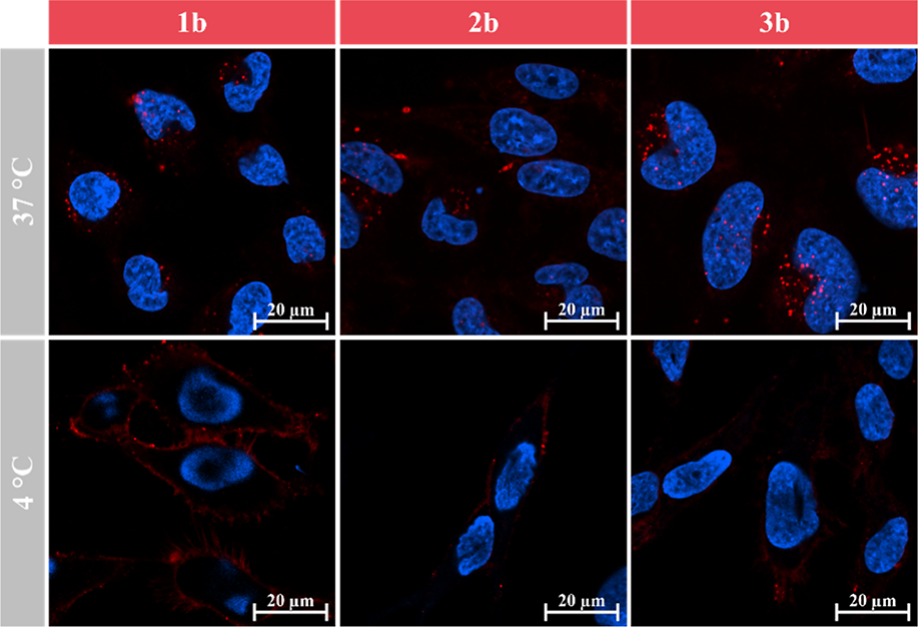
Live-cell CLSM images of U251-MG cells treated with compounds 1b, 2b, and 3b at 37 °C and at 4 °C, respectively. Images were recorded 15 min after incubation at 37 °C. Cell nuclei were stained with Hoechst 34580. Blue channel: Hoechst 34580, laser wavelength 405 nm; red channel: cy5, laser wavelength 639 nm. Laser power for cy5 was set to 2.4%.

**Figure 12:**
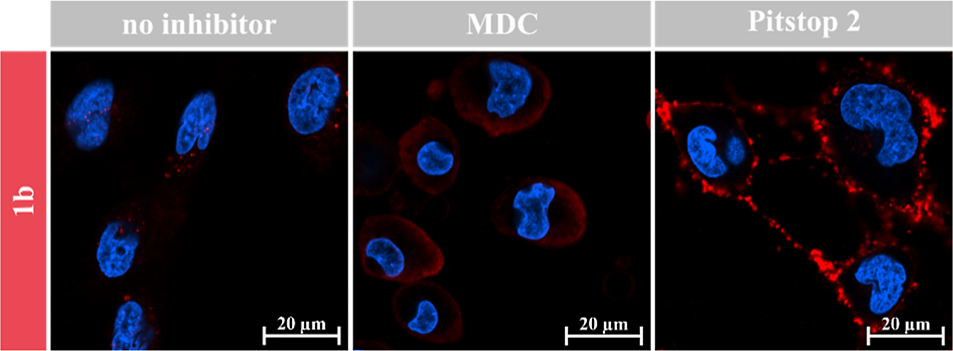
Live-cell CLSM images of U251-MG cells treated either solely with compound 1b or with prior preincubation with inhibitors MDC or Pitstop 2. Images were recorded 15 min after incubation at 37 °C. Cell nuclei were stained with Hoechst 34580. Blue channel: Hoechst 34580, laser wavelength 405 nm; red channel: cy5, laser wavelength 639 nm. Laser power for cy5 was set to 2.4%.

To gain insights into cellular uptake mechanisms, an additional set of uptake studies was carried out using U251-MG cells at 37 and 4 °C, respectively. CLSM images reveal that all investigated compounds (1b, 2b, and 3b) show uptake into the cells at 37 °C. Similar clusters of the compound as in the experiment with HEK293 cells were visible. In contrast, uptake experiments at 4 °C show predominant localization of the compound on the cell surface (Figure 11). The observed retention of polymers on the cell membranes under these conditions suggests an active process rather than passive diffusion, reinforcing the hypothesis that cellular machinery may be involved in the uptake process. This indicates that the attachment of the compounds to the cell surface via their stearyl group occurs as a passive process, while the uptake of the compounds into the cell is an active process that is suppressed at low temperatures. Further investigations explored the role of specific cellular pathways through the use of inhibitors. To exclude that the inhibition of cell uptake at 4 °C is caused by an increased rigidity of the cell membrane at this temperature,^85^ additional uptake studies with compound 1b as a representative structure and in the presence of inhibitors targeting clathrin-mediated endocytosis (MDC and PitStop 2) were carried out on U251-MG cells (Figure 12). In accordance with uptake experiments conducted at 4 °C, the compound predominantly remained on the cell surface. Notably, treatment with Pitstop 2 resulted in the compound refraining from internalization and instead forming clusters prominently on the cell surface. This result highlights the potential role of clathrin-mediated endocytosis in the internalization of hydrophobic, branched peptoids. Further, an uptake study in the presence of the phosphoinositide 3-kinase (PI3K) inhibitor LY294002 was carried out to suppress ATP-dependent processes in the cell. Despite inducing cellular damage, the internalization of polymers persisted, suggesting that PI3K may not be a key player in this process. Due to the potential impact of experiment-induced cell damage, CLSM images were not used for interpretation and are not shown.

**Figure 13:**
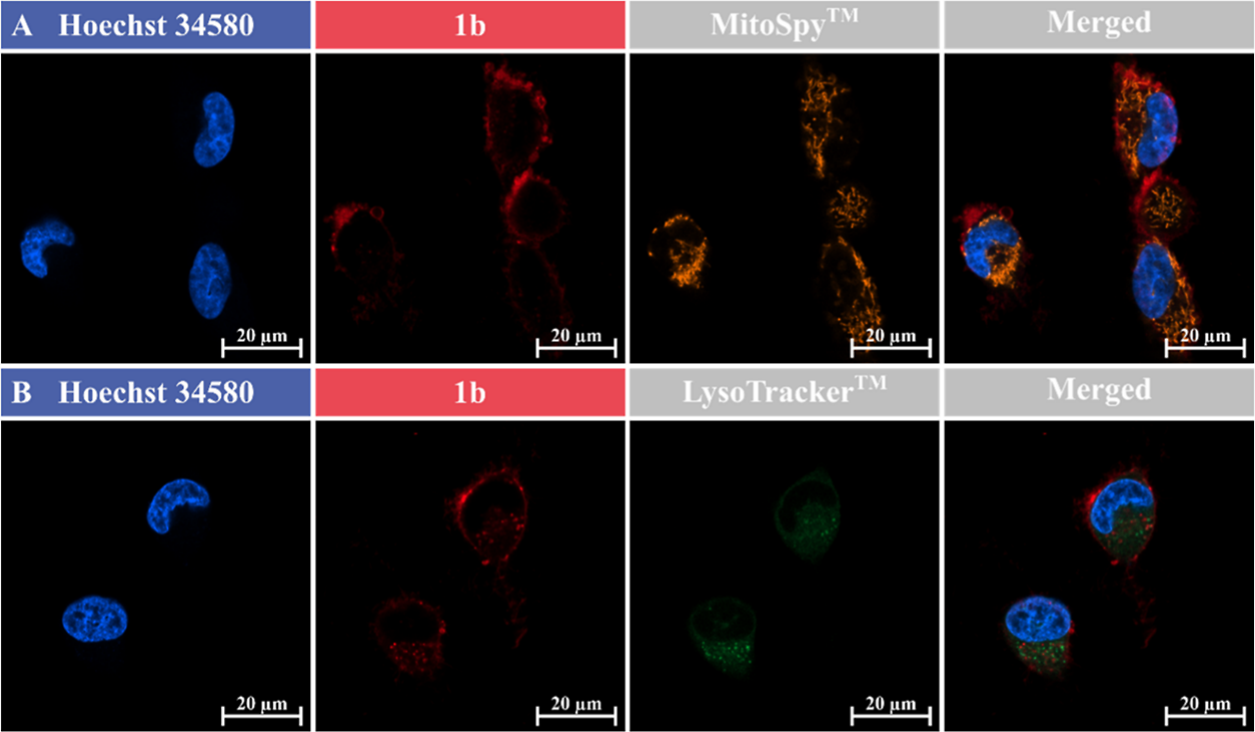
Live-cell CLSM images of U251-MG cells treated with compound 1b in the presence of MitoSpy Orange CMTMRos and LysoTracker Green DND-26, respectively. Cells were incubated with the mitochondria or lysosome staining reagent for 30 min, followed by incubation with compound 1b for 20 min. Cell nuclei were stained with Hoechst 34580. Imaging was performed at 37 °C using lasers with a wavelength of 405 nm for Hoechst 34580, 488 nm for LysoTracker, 561 nm for MitoSpy, and 639 nm for cy5. Laser power for cy5 was adjusted according to the respective dyes used for colocalization.

As the passive uptake of carboxylated, cy5-labeled polymers was reported to result in interactions between these compounds and mitochondria, colocalization studies were carried out on U251-MG cells using compound 1b and the mitochondrial localization probe MitoSpy Orange CMTMRos (Figure 13A). Analysis of CLSM images revealed no significant colocalization between the internalized compound 1b and mitochondria (Pearson correlation coefficient 0.27). Conversely, a parallel colocalization study on U251-MG cells, involving compound 1b and the fluorescent probe LysoTracker Green DND-26 for staining acidic compartments within cells, suggested colocalization of lysosomes and the internalized compound (Pearson correlation coefficient 0.52). This finding supports the assumption that the main uptake pathway of the investigated compounds is an endocytic, and therefore active, process (Figure 13B). The colocalization graphs used for the calculation of the Pearson correlation coefficients are shown in Figure S13.

In summary, we suggest that the compounds with stearyl end groups partially attach to, or intercalate into, the cell membrane in a passive process. This increases the local concentration of the compound at the cell surface and leads to an increased uptake of the latter via active processes (endocytosis). Uptake of compounds from the solution may occur as well, but is not prominent, which results in a low internalization of compounds without a hydrophobic anchor (compounds 1d, 2d and 3d), as they do not interact with the cell membrane. Compound 1d exhibits the lowest uptake, presumably due to its negative charge. Compounds 2b and 3b seem to be less likely to remain on the cell surface instead of being internalized when compared to compound 1b. This may be attributed to the observation that the total uptake appeared less significant compared to 1b. Additionally, internalization may be easier for uncharged compounds, which causes faster internalization of 2b and 3b after attachment of the compound to the cell membrane, while compound 1b is more likely to stay on the cell surface. These findings may prove the value of the generated structures for staining or targeting specific parts of the cell membrane, e.g., membrane-bound proteins, after functionalization with a suitable drug. Especially highly hydrophilic or charged drug molecules could be located close to their target on the cell membrane, while a certain mobility of the drug molecule is still given due to the hydrophilic fragments connecting drug and membrane anchor.

## 4 Conclusions

In this study, we synthesized branched polymeric structures based on OEG, PEG, and PEtOx fragments connected to an oligopeptoid backbone. Solid-phase synthesis was applied to generate compounds with an exactly defined number of side chains and additional functionalities, such as a reactive side group for the attachment of a fluorescent dye and a stearyl group as a hydrophobic anchor. We investigated the relationship between the structure of the compounds and their solution properties, as well as the consequent influence of the structure on the interaction with the HEK293 and U251-MG cells. In general, more hydrophilic structures led to a lower binding affinity for the substances. The stearyl group proved to enhance the nonspecific binding of the compounds to the cell. The resulting localization of a larger proportion of the compound close to the cell membrane appeared to increase its uptake by cells in comparison to compounds without a hydrophobic anchor. While higher concentrations of the compound influenced cell viability, no cell membrane disruption due to the attachment of the compounds to cells was detected for all oligopeptoids except for structure 1a. We prove that solid-phase synthesis may be an interesting tool to generate highly defined and functional polymeric structures, e.g., for medicinal chemistry, and the compounds presented in this manuscript are promising models or precursors for polymer-drug conjugates. Especially for targeting membrane-bound receptors, their ability to localize attached substances close to their target can be an advantage.

## Supporting Information Available

^1^H NMR spectra of compound 2b after attachment of cy5; detailed fluorescence excitation and emission map for compounds 1a–1d; fluorescence intensity–concentration plots used for determination of the CMC values; cytotoxicity study on the U251-MG cell line; results of hemolysis experiments for all compounds at different concentrations; degree of functionalization/effective concentration of compound stock solutions for biostudies; gating strategy for flow cytometry; CLSM images for compounds 2*a*/2d, 3*a*/3d, and untreated cells; and colocalization graphs for compound 1b and MitoSpy as well as LysoTracker (PDF)
