# Supplementary material for: Solid-Phase Synthesis as a Tool to Create Exactly Defined, Branched Polymer Vectors for Cell Membrane Targeting
Source: Macromolecules. 2024 Jan 26;57(3):1050–71. doi: 10.1021/acs.macromol.3c02600 (PMC10867888; doi:10.1021/acs.macromol.3c02600)
Supplement: Supplementary file 1 — ma3c02600_si_001.pdf [file ma3c02600_si_001.pdf]

Supporting Information

Solid-phase synthesis as a tool to create exactly defined, branched polymer vectors for cell membrane targeting

Johanna K. Elter[a], Veronika Liščáková[b, c], Oliver Moravec[a], Martina Vragović[a], Marcela Filipová[a], Petr Štěpánek[a], Pavel Šacha[b], Martin Hrubý[a*]*

[a] Institute of Macromolecular Chemistry, CAS

Heyrovského nám. 2, 162 06 Praha 6, Czech Republic

[b] Institute of Organic Chemistry and Biochemistry, CAS

Flemingovo nám. 2 166 10 Praha 6, Czech Republic

[c] First Faculty of Medicine, Charles University

Kateřinská 1660/32, 121 08 Praha 2, Czech Republic

* johanna.elter@gmx.de, martin.hruby@centrum.cz

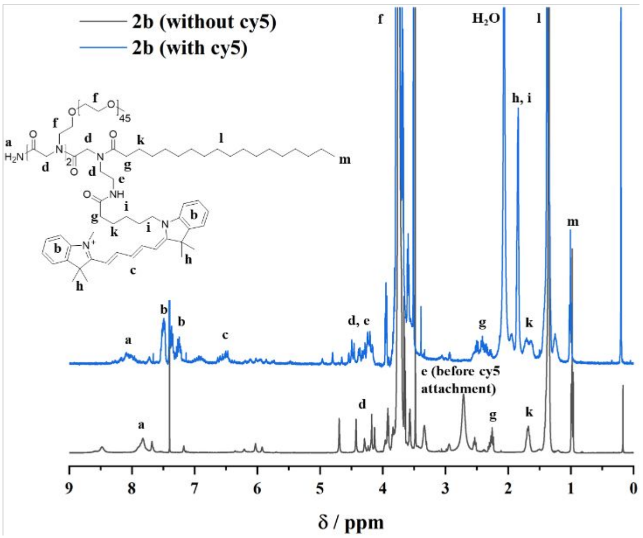

Figure S1. Compound 2b before (grey) and after (blue) attachment of cyanine 5 (cy5) active ester as a fluorescent label. ^{1}H NMR spectra in the manuscript do not contain fluorescent dye, as the amount of labeled compound generated was insufficient to generate spectra of sufficient resolution.

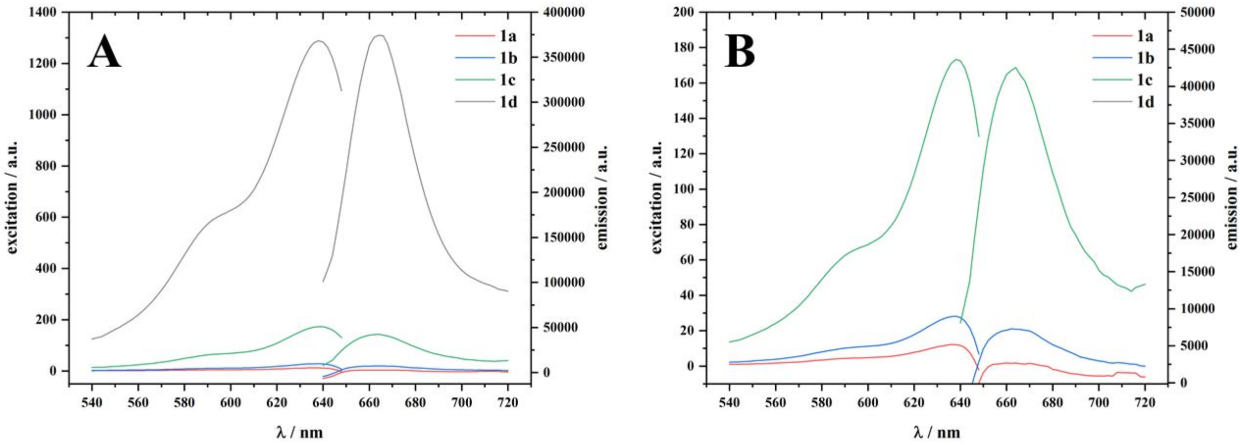

Figure S2. Fluorescence excitation and emission maps of compounds 1a – 1d as shown in the manuscript (A), and magnified to display the effect of fluorescence quenching due to the aggregation of compounds 1a – 1c (B). Excitation maps were measured at a detection wavelength of 660 nm, while emission spectra were measured at an excitation wavelength of 638 nm. The concentration of the compounds was set to 10 µg mL^{-1} to obtain reasonable absorbance and emission values.

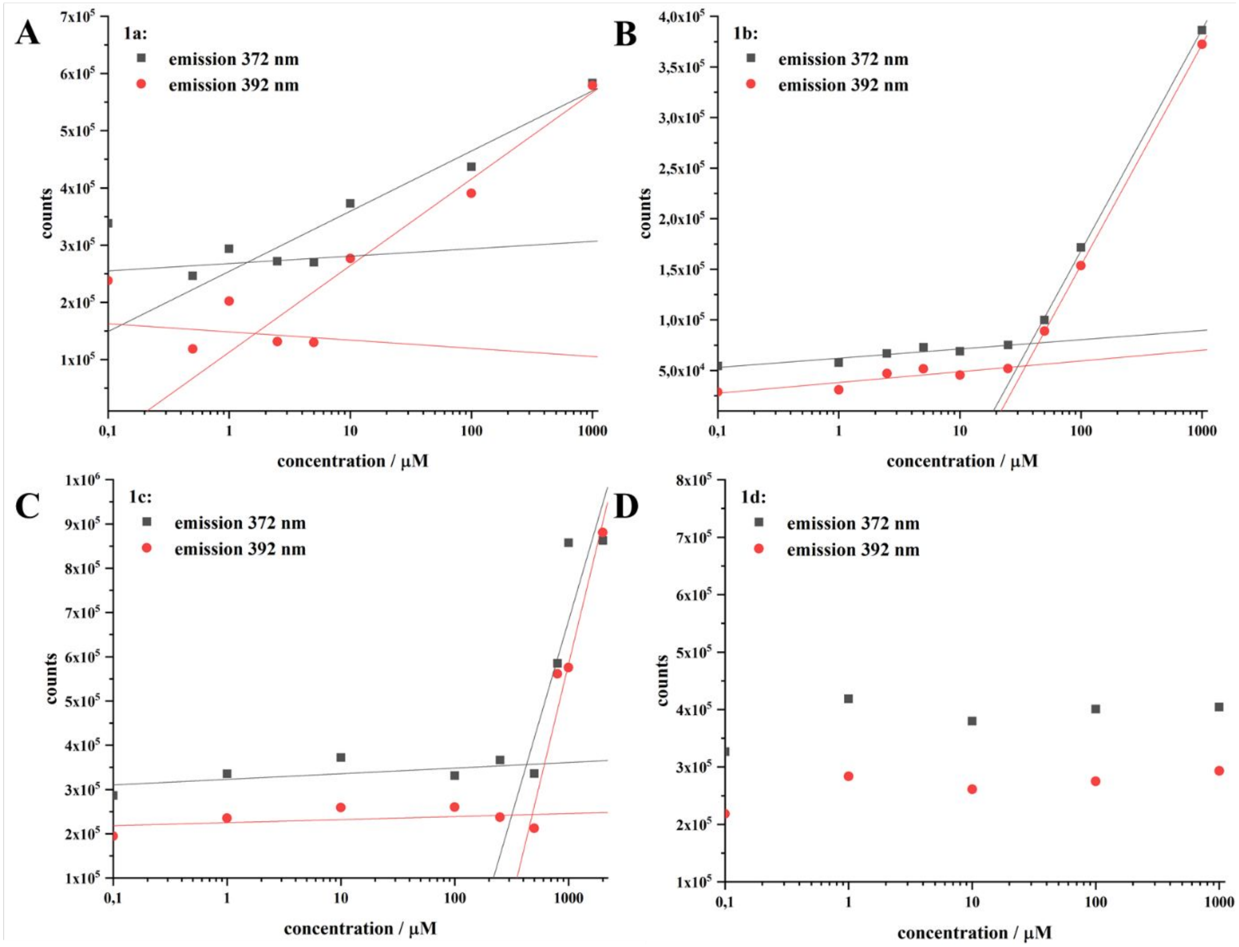

Figure S3. Plots of pyrene fluorescence versus oligopeptoid concentration for determination of the critical micelle concentration of compounds 1a – 1d in PBS.

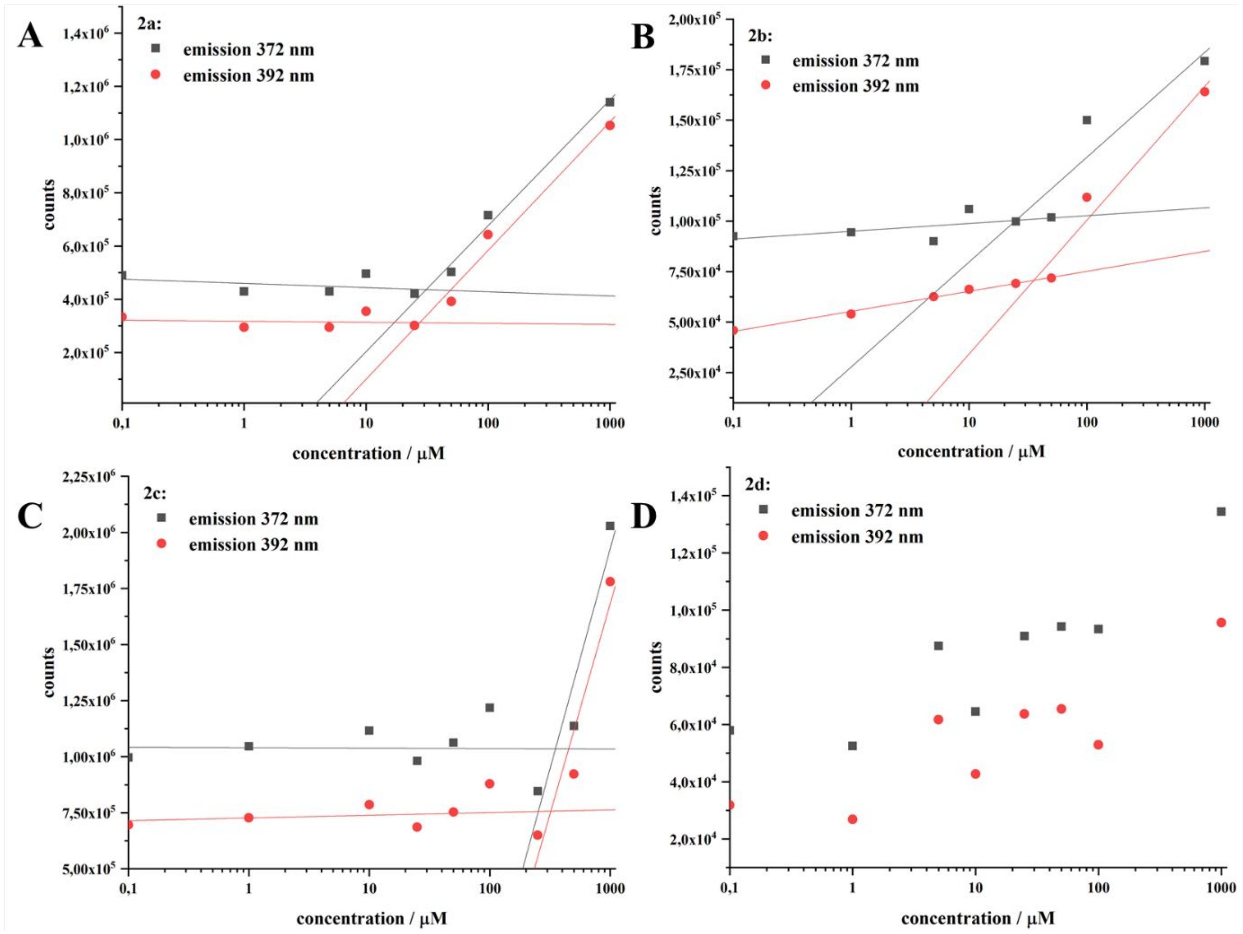

Figure S4. Plots of pyrene fluorescence versus oligopeptoid concentration for determination of the critical micelle concentration of compounds 2a – 2d in PBS.

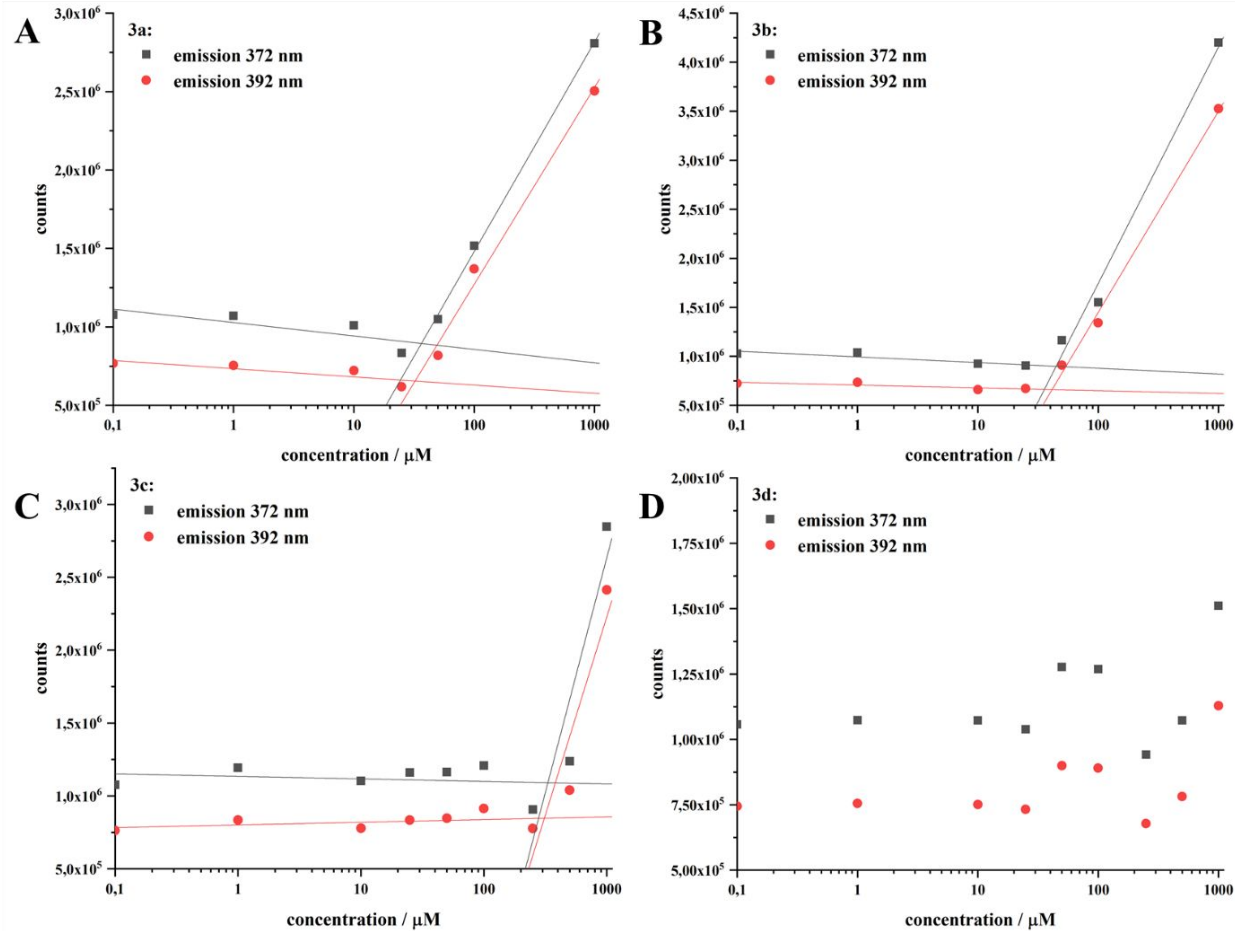

Figure S5. Plots of pyrene fluorescence versus oligopeptoid concentration for determination of the critical micelle concentration of compounds 3a – 3d in PBS.

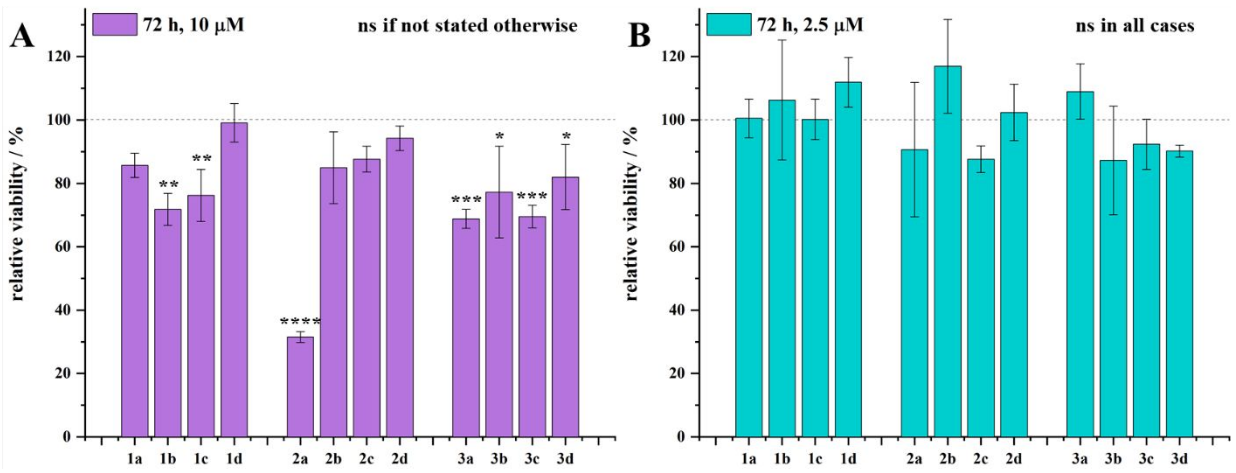

Figure S6. Relative metabolic activity of the U251-MG cells, normalized to the untreated control, as determined by probing the ATP production of the cells *via* a luminescent-based assay at a concentration of 10 μM (A) and 2,5 μM (B). The study was carried out for 72 h.

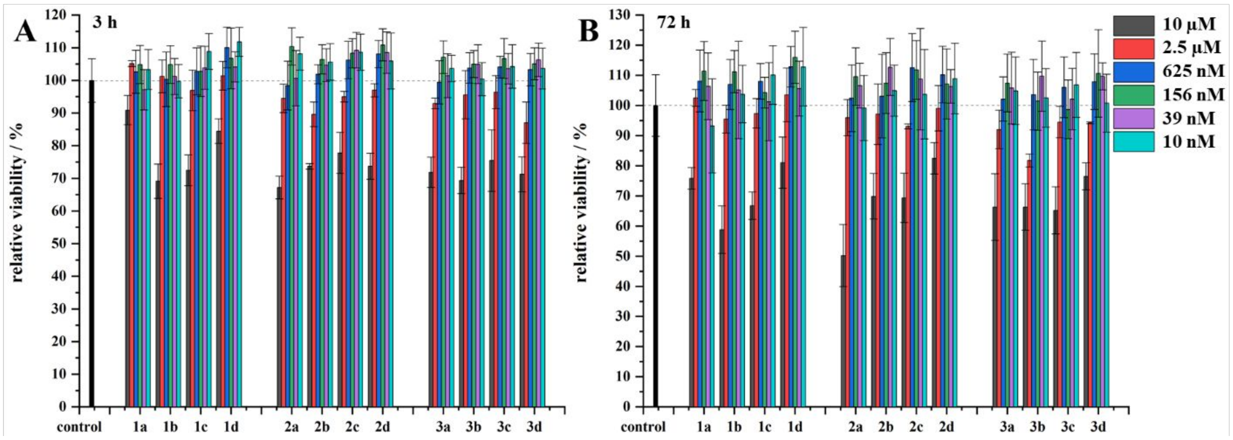

Figure S7. Relative metabolic activity of the HEK293 cells, normalized to the respective untreated control in each assay, as determined by probing the ATP production of the cells *via* a luminescent-based assay in a concentration range from 10 μM to 10 nM. The study was carried out for 3 h (A) or 72 h (B), respectively.

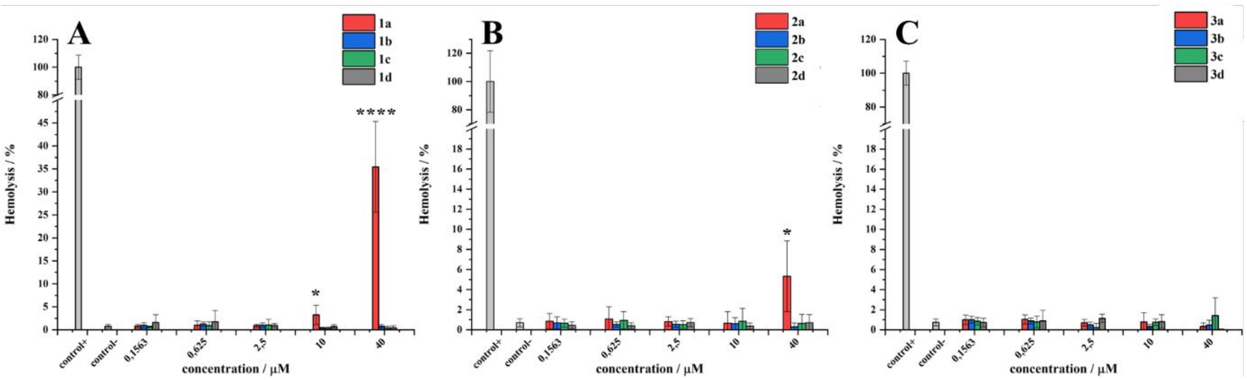

Figure S8. Hemolysis (%) of human RBCs caused by compounds 1a – d (A), 2a – d (B), and 3a – d (C) depending on their concentration. control+: 1% Triton X-100; control-: PBS.

Table S1. Degree of functionalization of the oligopeptoids with the cy5 dye (attached to the free amine group using the cy5-TT active ester) as determined by ^{1}H NMR spectroscopy in $CDCl_3$ as well as UV/vis spectroscopy of a 5 µmol solution the compounds in EtOH. For compounds with a degree of functionalization of 1.00 in ^{1}H NMR, the spectra showed full conversion within the error range of the method. For compound 3d, the water signal in the ^{1}H NMR spectrum did not allow integration of the respective peaks. MALDI-TOF MS spectroscopy further proved full functionalization of compounds 1a – d.

**Compound**	**Degree of functionalization with cy5 (^{1}H NMR)**	**Degree of functionalization with cy5 (UV/vis)**
**1a**	1.00	0.23
**1b**	1.00	1.00
**1c**	1.00	0.97
**1d**	1.00	0.46
**2a**	1.00	0.98
**2b**	0.92	0.74
**2c**	1.00	0.82
**2d**	0.58	0.56
**3a**	0.84	0.67
**3b**	0.78	0.94
**3c**	1.00	0.58
**3d**	n.d.	0.67

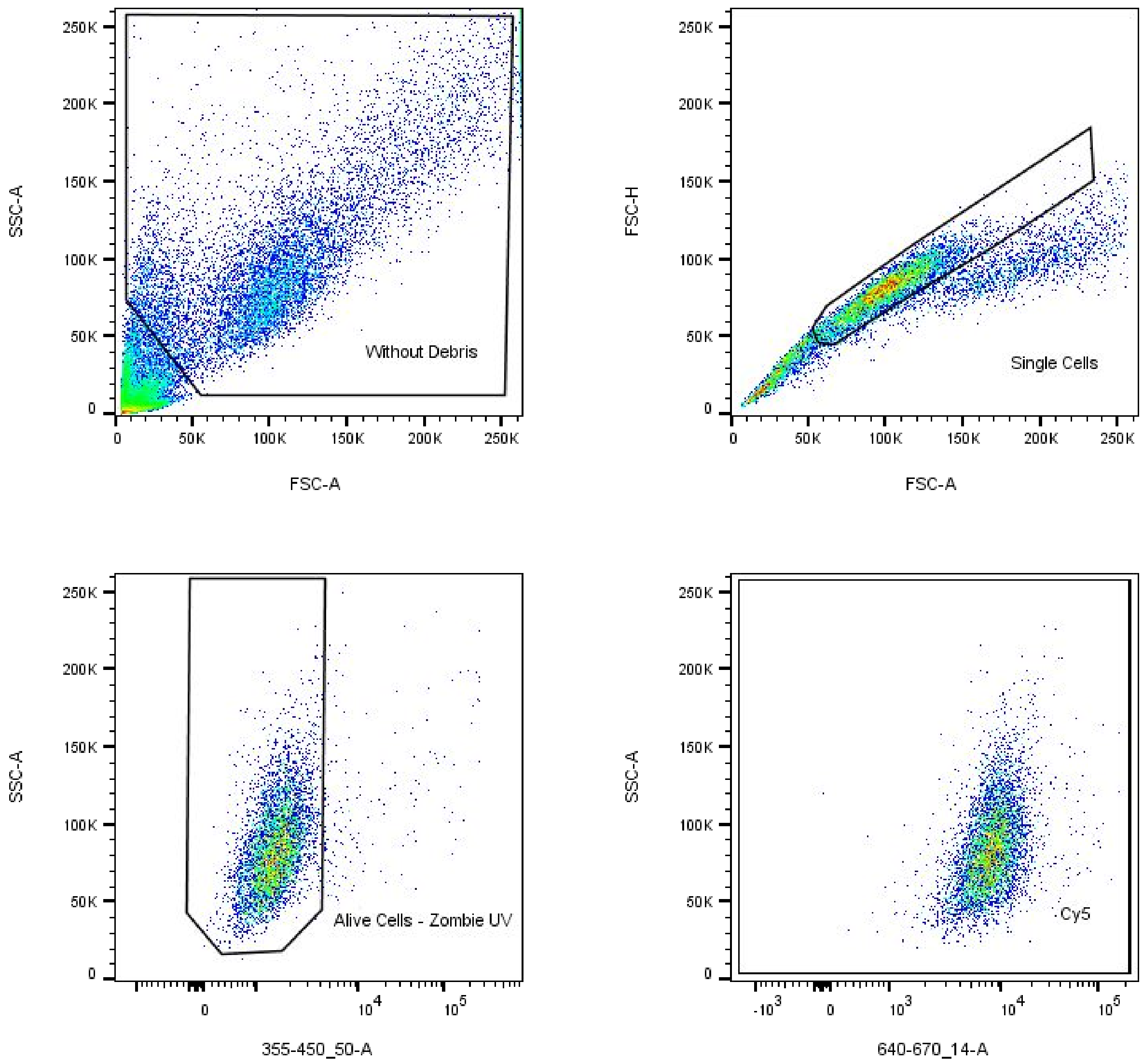

Figure S9. Gating strategy as applied in the FlowJo™ v10 Software to analyze flow cell cytometry results. The figure illustrates HEK293 population being subgated to the level of cy5-labeled cells.

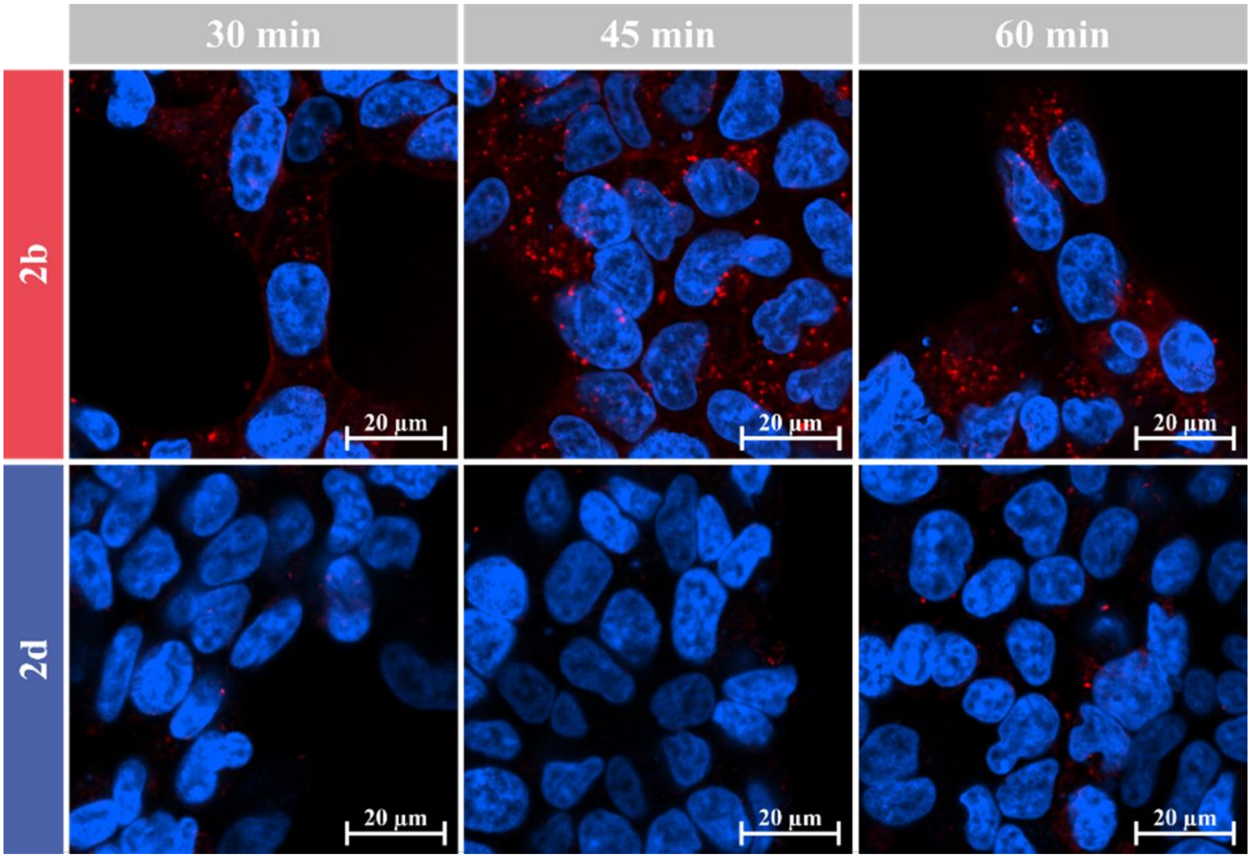

Figure S10. Live-cell CLSM images of HEK293 cells treated with compounds 2b (with hydrophobic anchor) and 2d (without hydrophobic anchor), recorded at different times after a 20 min incubation of the cells with respective compounds. Increased uptake of compound 2b in comparison to compound 2d was observed. Membrane staining was visible, but less prominent compared to compound 2b. Cell nuclei were stained with Hoechst 34580. Blue channel: Hoechst 34580, laser wavelength 405 nm; red channel: cy5, laser wavelength 639 nm. Laser power for cy5 was set to 3 %.

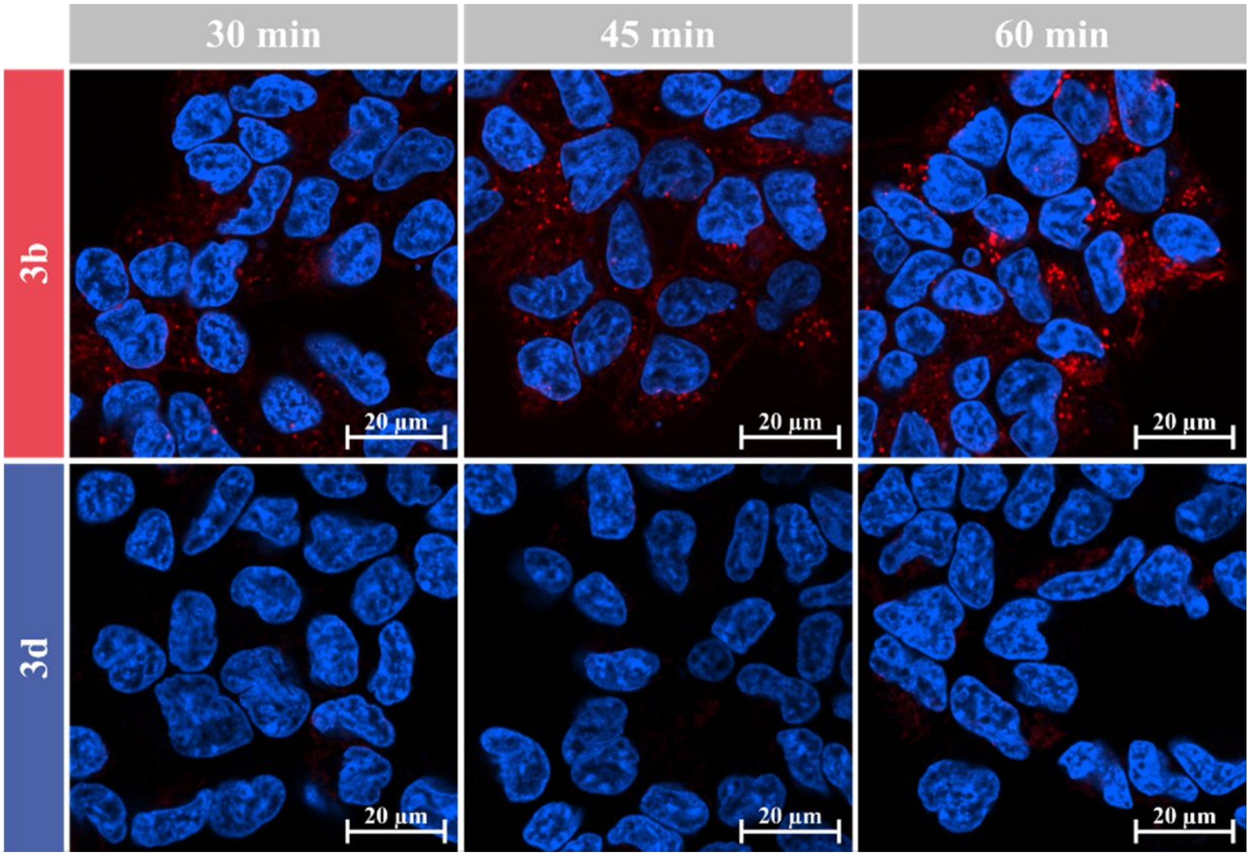

Figure S11. Live-cell CLSM images of HEK293 cells treated with compounds 3b (with hydrophobic anchor and 3d (without hydrophobic anchor), recorded at different times after a 20 min incubation of the cells with respective compounds. Again, increased uptake of compound 3b in comparison to compound 3d was observed. The behavior of the compounds in cell incubation experiments was comparable to the behavior of compounds 2b and 2d, respectively. Cell nuclei were stained with Hoechst 34580. Blue channel: Hoechst 34580, laser wavelength 405 nm; red channel: cy5, laser wavelength 639 nm. Laser power for cy5 was set to 3 %.

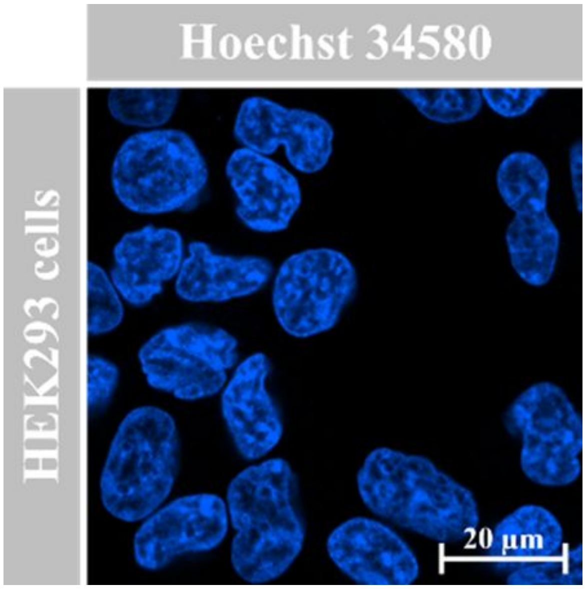

Figure S12. Live-cell CLSM image of untreated HEK293 cells. The cell nuclei only were stained with Hoechst 34580 to check for autofluorescence. Blue channel: Hoechst 34580, laser wavelength 405 nm; red channel: cy5, laser wavelength 639 nm. Laser power for cy5 was set to 3 %.

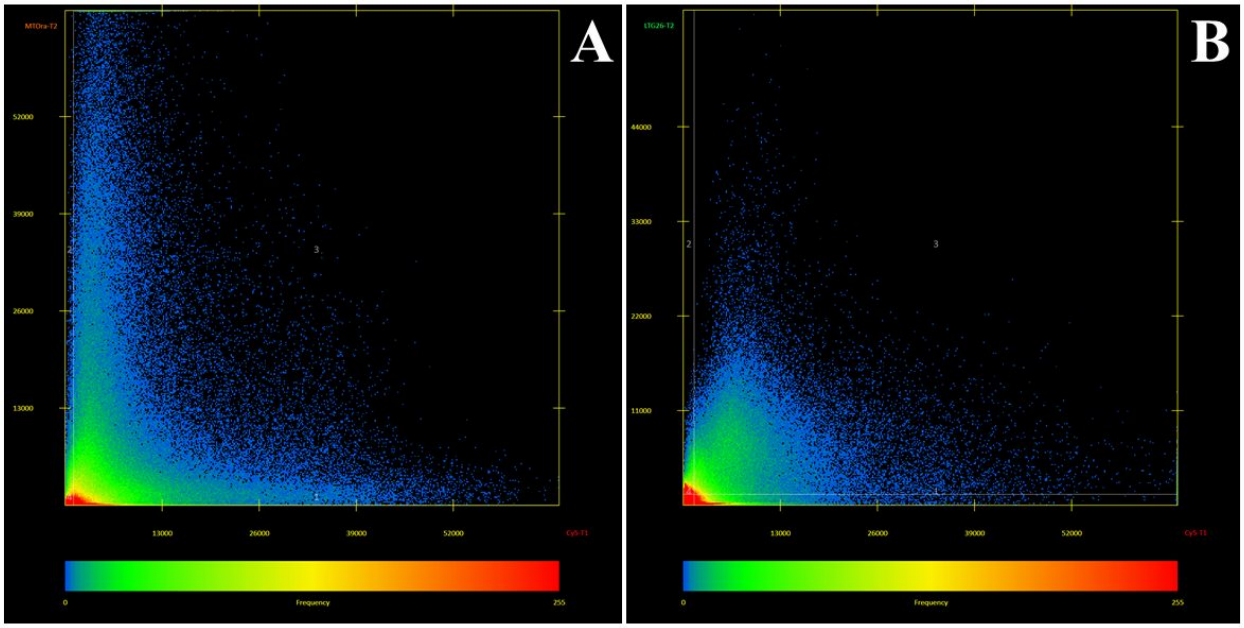

Figure S13. Colocalization graphs for MitoSpy™ Orange CMTMRos (A) and LysoTracker™ Green DND-26 (B) with compound 1b in cell colocalization experiments on U251-MG cells. Cells were incubated with the mitochondria or lysosome staining reagent for 30 min, followed by incubation with compound 1b for 20 min. Cell nuclei were stained with Hoechst 34580. Imaging was performed at 37 °C using lasers with a wavelength of 405 nm for Hoechst 34580, 488 nm for LysoTracker, 561 nm for MitoSpy, and 639 nm for cy5. Pearson correlation coefficient for the colocalization of MitoSpy™ Orange CMTMRos and 1b: 0.27. Pearson correlation coefficient for the colocalization of LysoTracker™ Green DND-26 and 1b: 0.52.
